# An intracellular complement system drives metabolic and proinflammatory reprogramming of vascular fibroblasts in pulmonary hypertension

**DOI:** 10.1172/jci.insight.184141

**Published:** 2025-02-13

**Authors:** Ram Raj Prasad, Sushil Kumar, Hui Zhang, Min Li, Cheng-Jun Hu, Suzette Riddle, Brittany A. McKeon, M.G. Frid, Konrad Hoetzenecker, Slaven Crnkovic, Grazyna Kwapiszewska, Rubin M. Tuder, Kurt R. Stenmark

**Affiliations:** 1 Cardiovascular and Pulmonary Research Laboratory (CVP), Department of Pediatrics and Medicine, and; 2 Department of Craniofacial Biology, University of Colorado, Anschutz Medical Campus, Aurora, Colorado, USA,; 3 Department of Thoracic Surgery, Medical University of Vienna, Vienna, Austria.; 4 Ludwig Boltzmann Institute for Lung Vascular Research, Otto Loewi Research Center, Lung Research Cluster, Medical University of Graz, Graz, Austria.; 5 Institute for Lung Health, Cardiopulmonary Institute, Member of the German Center for Lung Research, Justus Liebig University Giessen, Germany.; 6 Department of Lung Biology, University of Colorado, Anschutz Medical Campus, Aurora, Colorado, USA.

**Keywords:** Pulmonology, Vascular biology, Cardiovascular disease, Complement

## Abstract

The complement system is central to the innate immune response, playing a critical role in proinflammatory and autoimmune diseases such as pulmonary hypertension (PH). Recent discoveries highlight the emerging role of intracellular complement, or the “complosome,” in regulating cellular processes such as glycolysis, mitochondrial dynamics, and inflammatory gene expression. This study investigated the hypothesis that intracellular complement proteins C3, CFB, and CFD are upregulated in PH fibroblasts (PH-Fibs) and drive their metabolic and inflammatory states, contributing to PH progression. Our results revealed a pronounced upregulation of CFD, CFB, and C3 in PH-Fibs from human samples and bovine models, both in vivo and in vitro. The finding of elevated levels of C3 activation fragments, including C3b, C3d, and C3a, emphasized enhanced C3 activity. PH-Fibs exhibited notable metabolic reprogramming and increased levels of proinflammatory mediators such as MCP1, SDF1, IL-6, IL-13, and IL-33. Silencing CFD via shRNA reduced CFB activation and C3a production, while normalizing glycolysis, tricarboxylic acid (TCA) cycle activity, and fatty acid metabolism. Metabolomic and gene expression analyses of CFD-knockdown PH-Fibs revealed restored metabolic and inflammatory profiles, underscoring CFD’s crucial role in these changes. This study emphasizes the crucial role of intracellular complement in PH pathogenesis, highlighting the potential for complement-targeted therapies in PH.

## Abstract 2

Increased expression and activation of intracellular complement (C3, CFB, and CFD) in adventitial fibroblasts leads to metabolic and inflammatory changes that contribute to the pathogenesis of pulmonary hypertension.

## Graphical abstract

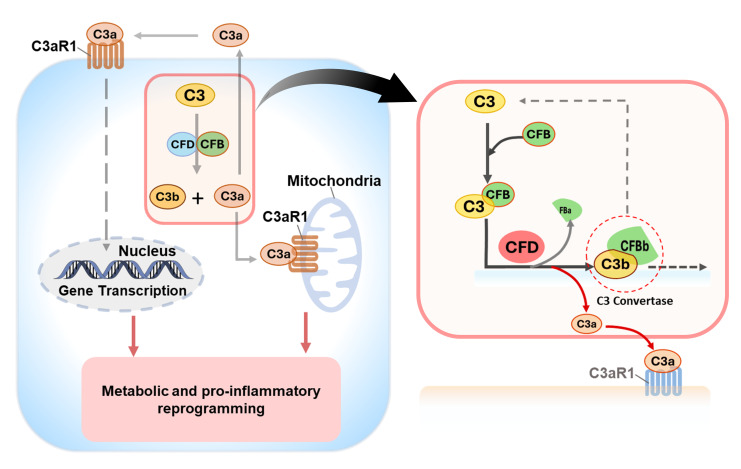

## Introduction

Pulmonary hypertension (PH) is a prevalent cardiopulmonary disorder characterized by pulmonary vascular remodeling, leading to increased pulmonary artery (PA) pressure and pulmonary vascular resistance. This progressive remodeling culminates in right heart functional decline and, ultimately, failure (1, 2). Central to pulmonary vascular remodeling are hallmarks such as excessive cellular proliferation, extracellular matrix deposition, and immune cell accumulation within the PA (3). In recent years, inflammation has emerged as a critical driver of PH, with the immune system increasingly recognized as a key player in its pathobiology (4).

Using advanced digital spatial profiling to analyze vascular lesions in IPAH pathology, we observed that inflammation-related pathways, including interferon signaling, TNF signaling, allograft rejection, hypoxia, and IL-2/IL-6/Jak-Stat signaling, were enriched in adventitial and plexiform lesions. This finding aligns with growing evidence implicating fibroblast-mediated inflammation in the pathogenesis of PH (5). Our data position the adventitia as a hub for immune mechanisms in idiopathic pulmonary arterial hypertension (IPAH). Consistent with this, our prior reports suggest C3 activation in the adventitia of patients with IPAH and various PH animal models (6). We documented elevated transcript expression of several classical and alternative complement cascade members (C1Q, C1S, C7, C3, complement factor D [CFD], and the regulatory protein CFH) in IPAH adventitia compared with other pulmonary vascular lesions (5).

It is now well understood that complement function is highly compartmentalized and operates systemically, locally within extracellular spaces, and intracellularly within subcellular compartments and organelles. This intricate system profoundly influences inflammation, contributing to numerous inflammatory diseases (7). Despite its importance, the role of the complement system — particularly the intracellular complement system, or “complosome” — in fibroblasts and its contribution to PH, in which inflammation is an important pathological driver, remains inadequately explored (7, 8).

Within immune cells such as T cells, B cells, neutrophils, and macrophages, the complosome plays a pivotal role in regulating cellular homeostasis and effector functions (9–11). Intriguingly, intracellular complement activities extend beyond immune cells to nonimmune cells, including epithelial, endothelial cells (ECs), mesothelial cells, and fibroblasts (12–16). Recent findings in cultured cells suggest that PA fibroblasts are a prominent source of complement components, including C3, CFB, and CFD. Intracellular activation of C3 and interaction of C3 activated component C3b with CD46 are known to activate mTOR nutrient-sensing pathways and glucose uptake (17, 18). Additionally, complement C3a receptor 1 (C3aR1) and complement C5a receptor 1 (C5aR1), localized on the outer mitochondrial membrane, regulate the balance between ROS production and ATP generation, thereby influencing cellular metabolism (14, 19). Dysregulation of complosome activities has been implicated in numerous human diseases, including recurrent infections, arthritic conditions, atherosclerosis, and cancer (13, 20–22).

In this study, we hypothesized that expression and activation of the complosome, particularly through the alternative pathway, are critical for driving the persistently activated proinflammatory phenotype of fibroblasts in the PAs of individuals with PH. To test this hypothesis, we utilized primary cultured PA fibroblasts that were isolated from patients with IPAH and bovine calves with severe hypoxia-induced PH (PH-Fibs), as well as corresponding controls (CO-Fibs), as in vitro model systems. We performed comprehensive evaluations of complement alternative pathway components in fibroblasts in vivo and in vitro. To investigate the role of intracellular CFD in the activation of C3, metabolic reprogramming, and proinflammatory phenotypes in adventitial fibroblasts, we employed gene manipulation techniques, pharmacologic blockade, and protein-protein interaction analysis, transcripts analysis, and mass spectrometry–based (MS-based) metabolomics analysis. Our findings reveal that CFD-driven activation of C3 and its fragments modulates metabolic functions and the proinflammatory phenotype of adventitial fibroblasts.

## Results

### In vivo, PA fibroblasts in PAs of IPAH patients and PH calves exhibit elevated expression of C3, CFB, and CFD.

**Figure 1:**
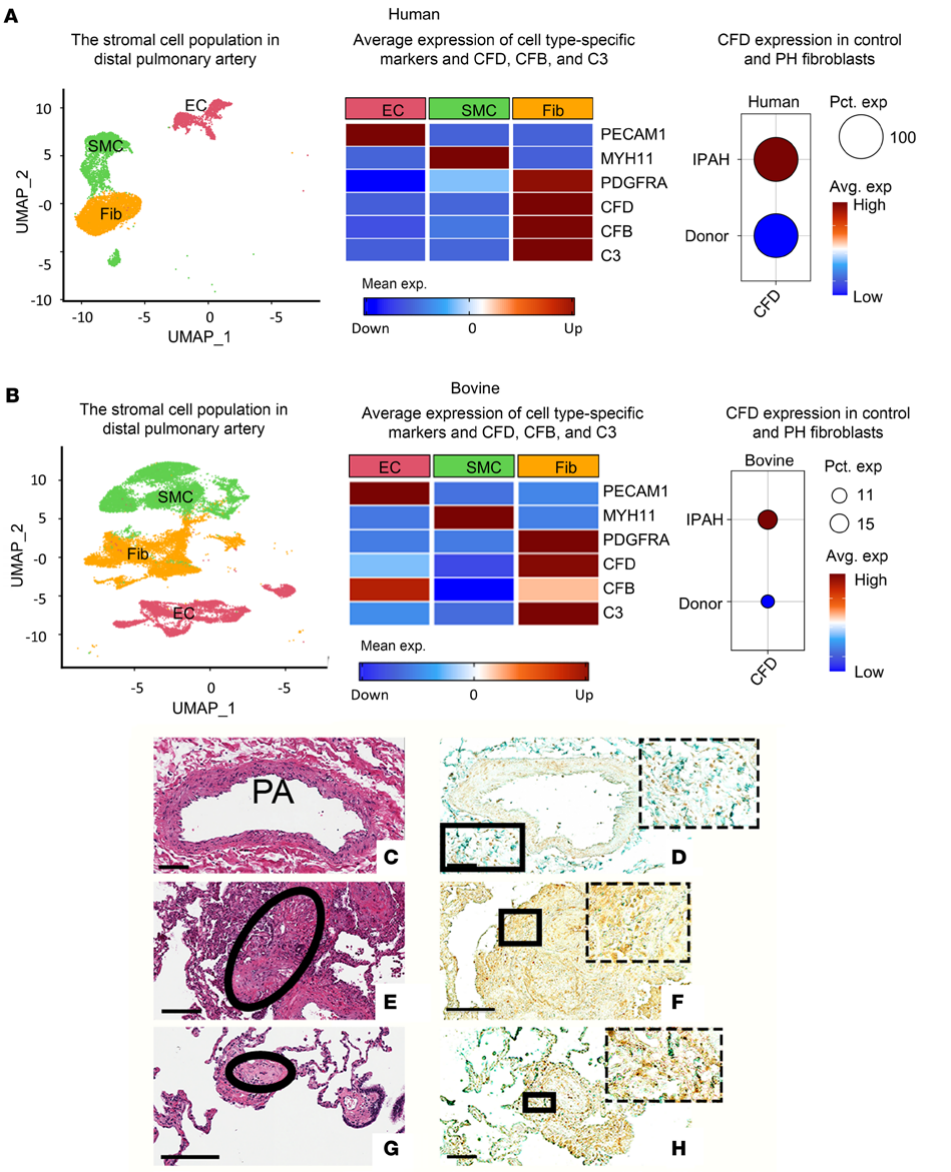
In vivo, PA fibroblasts in PAs of patients with IPAH and PH calves exhibit elevated expression of C3, CFB, and CFD. (**A**) Left: UMAP visualization of human stromal cells: ECs *n* = 1,753, SMCs *n* = 3,126, and Fibs *n* = 4,523. Middle: Average expression of cell-specific markers and CFD. Right: Percentage of cells (Pct. exp) and average CFD gene expression (Avg. exp) in human PA fibroblasts. Source of human data: ref. 23. (**B**) Left: UMAP visualization of bovine stromal cells: ECs *n* = 6,582, SMCs *n* = 10,901, and Fibs *n* = 1,016. Middle: Average expression of cell-specific markers, including PECAM1 for ECs, MYH11 for SMCs, PDGFRA for fibroblasts, and CFD. Right: Percentage of cells and average CFD gene expression in bovine PA fibroblasts. Immunohistochemical staining of serial sections of PAs from control PA (**D**) or IPAH lungs (**F** and **H**) with H&E (**C**, **E**, and **G**) or CFD. (**D**) The inset (dashed rectangle) shows a high-magnification image of the rectangular area of the adventitia of the control PA, with low expression of CFD. (**E** and **F**) The plexiform lesion is outlined by the circle. The dashed inset shows the magnification of the cellular core in the rectangular outline, with high expression of CFD (**F**). (**G** and **H**) IPAH obliterative lesion (circled area in **G**). The dashed inset shows a high magnification of the boxed area, with adventitia cells, largely fibroblasts, expressing high levels of CFD. Staining protocols were as reported in ref. 5. Scale bars: **C**, **D**, and **H**, 100 μm; **E**–**G**, 200 μm. scRNA-Seq data are presented for bovine (*n* = 3 control animals, *n* = 2 PH animals) and participants (*n* = 3 healthy donors, *n* = 3 with IPAH).

To evaluate both basal and altered expression of complement-associated genes such as C3, CFB, and CFD in PAs in vivo, we analyzed single-cell RNA-Seq (scRNA-Seq) data generated from PA cells in patients with PH (23) and PH calves, a robust large-animal model of PH (24), along with their respective controls. Our analysis concentrated on stromal cells due to their reported role in inflammatory PH remodeling (25–27). Clustering analysis of stromal cells revealed 3 distinct clusters, identified as ECs, smooth muscle cells (SMCs), and fibroblasts, based on the expression of canonical cell type–specific markers (Figure 1, A and B). For instance, mean expression of cell type–specific markers, such as PECAM1, MYH11, and PDGFRA, was high in ECs, SMCs, and fibroblasts, respectively.

Interestingly, we observed substantially higher expression of complement factors C3, CFB, and CFD in vascular fibroblasts (Figure 1, A and B). Specifically, we found elevated expression of CFD in both human IPAH-Fibs and bovine PH-Fibs compared with their respective control groups. However, in bovine PAs, not all fibroblasts expressed CFD; only specific populations exhibited CFD expression. These in vivo data demonstrated that among the PA stromal cell types expressing complement factor mRNAs, such as ECs and SMCs, the fibroblasts had the highest levels.

The increased expression of the CFD protein in IPAH aventitia compared with the control was further confirmed by immunohistochemical staining of PAs of patients with IPAH (Figure 1, C–H), consistent with previously reported quantitative finding (5).

### In vitro, primary cultured PA adventitial fibroblasts from IPAH patients and PH calves exhibit elevated expression of complement genes and increased production of activated C3 fragments.

**Figure 2:**
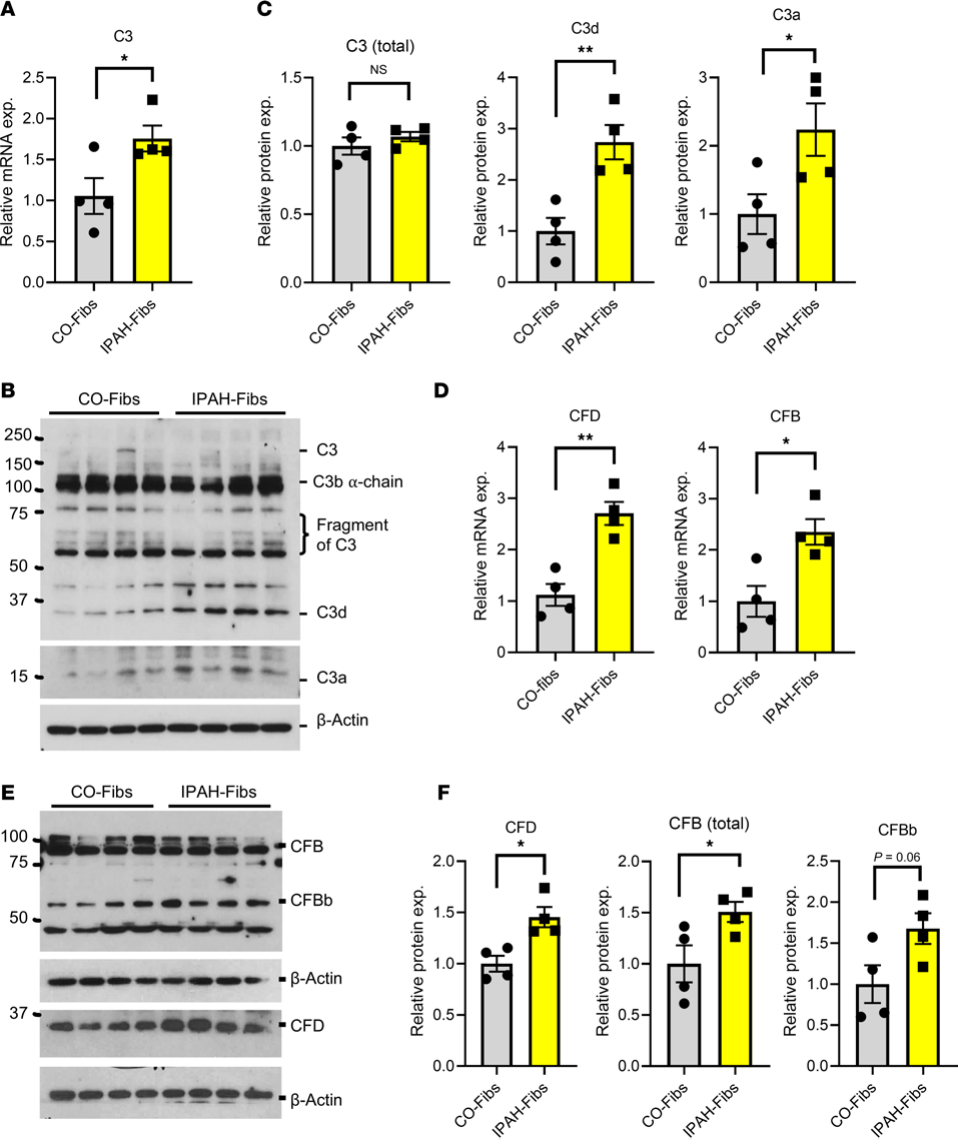
Human IPAH fibroblasts exhibit elevated expression of complement genes (C3, CFD, and CFB) and increased production of activated C3 fragments (C3d and C3a). (**A**) qRT-PCR data showing mRNA expression (exp.) levels of C3 in control and IPAH fibroblasts. (**B**) Western blot and (**C**) corresponding quantification data show C3, C3d, and C3a protein levels in control and IPAH fibroblasts. (**D**) qRT-PCR data indicate mRNA expression levels of CFB and CFD in control and IPAH fibroblasts. (**E**) Western blot and (**F**) relative protein quantification data of CFD and CFB in control and IPAH fibroblasts. Unpaired 2-tailed *t* test was used to compare 2 groups of samples. Data are presented as mean ± SEM from human control and IPAH fibroblast; *n* = 4. *P ≤ 0.05, **P < 0.01.

Primary cultured adventitial fibroblasts, isolated from human IPAH and bovine PH samples, recapitulated several key features of PH-Fibs observed in vivo (25–27). To avoid the deposition and effects of exogenous serum complements, we cultured the human IPAH, bovine PH, and control PA fibroblasts under serum-free conditions. Interestingly, we found that while mRNA levels of C3 were significantly increased in IPAH-Fibs, this did not correspond to a significant increase in total C3 protein expression (total C3 included all detected C3 and C3 fragments) (Figure 2, A–C). However, marked increases in activated C3 fragment proteins, including C3d and C3a, were observed in IPAH-Fibs, indicative of complement activation (Figure 2, B and C). Significant increases in CFD and CFB mRNA levels were also noted in IPAH-Fibs (Figure 2D). Additionally, increased levels of CFD, CFB, and CFBb (activated fragment of CFB) proteins were confirmed in IPAH-Fibs (Figure 2, E and F).

**Figure 3:**
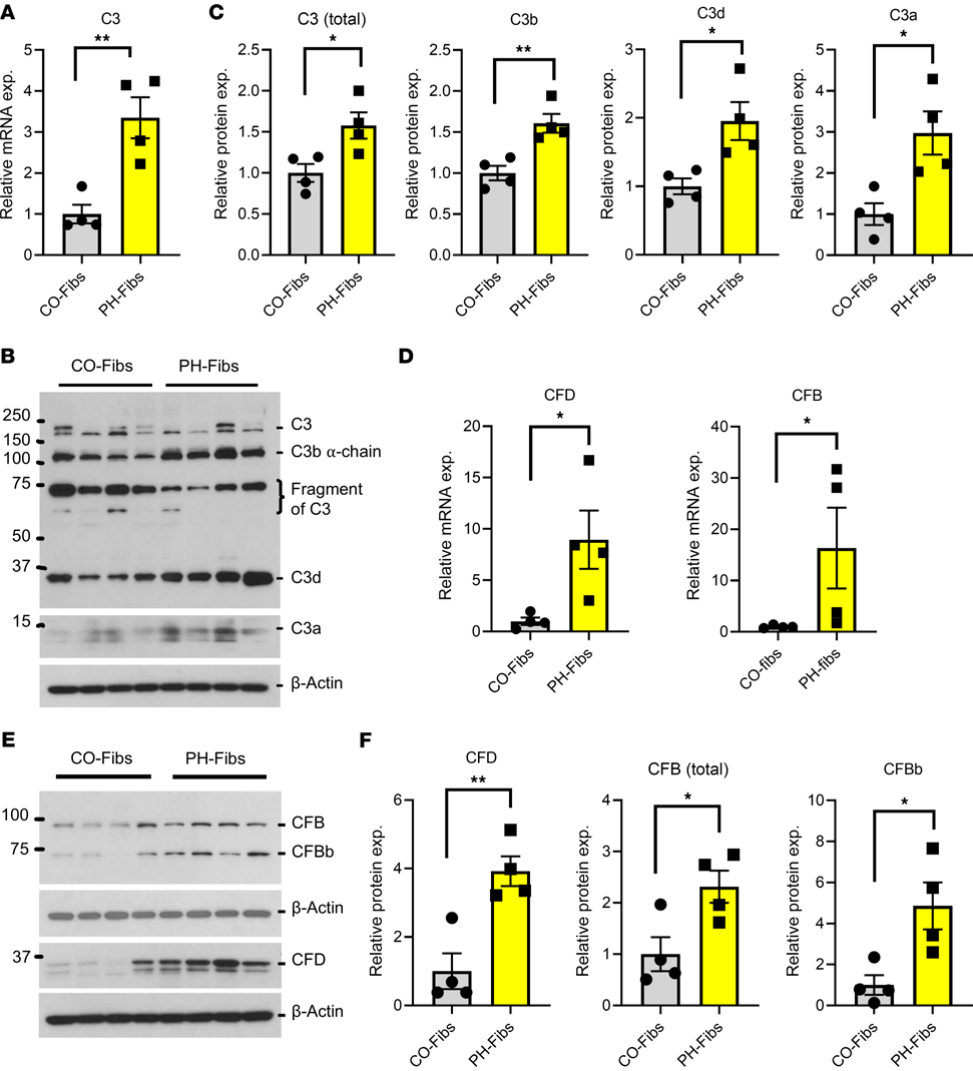
Bovine PH-Fibs show increased expression of complement genes (C3, CFD, and CFB) and increased production of activated C3 fragments (C3b, C3d, and C3a). (**A**) qRT-PCR data show the mRNA expression levels of C3 in bovine control and PH adventitial fibroblasts. (**B**) Western blot and (**C**) corresponding quantification data represent the protein levels of C3 and its activated peptides (C3d and C3a) in bovine control and PH adventitial fibroblasts. (**D**) qRT-PCR data indicate the mRNA expression levels of CFB and CFD in bovine control and PH adventitial fibroblasts. (**E**) Western blot and (**F**) relative protein quantification data of CFD and CFB in bovine control and PH adventitial fibroblasts. Unpaired 2-tailed *t* test was used to compare 2 groups of samples. Data are presented as mean ± SEM from bovine CO- and PH-Fibs; *n* = 4. *P ≤ 0.05, **P < 0.01.

Similarly, significant increases in C3 mRNA and total protein expression (total C3 included all detected C3 and C3 fragments) levels were observed in PH-Fibs cultured from young calves (15-28 days at the time of sacrifice) with severe hypoxia-induced PH compared with fibroblasts cultured from PAs of age-matched control calves (CO-Fibs) (Figure 3, A–C). Increased levels of activated C3 fragment proteins, including C3b, C3d, and C3a, were found in bovine PH-Fibs, confirming intracellular activation (Figure 3, B and C). Strikingly, consistent with the findings in humans, the PH-Fibs displayed marked increases in CFD and CFB mRNA expression (Figure 3D). Elevated protein levels of CFD, CFB, and CFBb were also noted in PH-Fibs (Figure 3, E and F).

The increased expression of the C3, CFD, and CFB genes in bovine PH-Fibs was further supported by assay for transposase-accessible chromatin (ATAC) sequencing data, which revealed higher levels of open chromatin structure in PH-Fibs compared with CO-Fibs (Supplemental Figure 1, A–C; supplemental material available online with this article; https://doi.org/10.1172/jci.insight.184141DS1). These results align with the increased expression of CFD, CFB and C3 observed in PA adventitial fibroblasts of patients with IPAH and PH calves using single-cell sequencing analysis (Figure 1). Collectively, these findings demonstrate the marked upregulation of the complement alternative pathway in human and bovine fibroblasts under pulmonary hypertensive conditions.

### CFD regulates the activation of C3 in PA adventitial fibroblasts.

**Figure 4:**
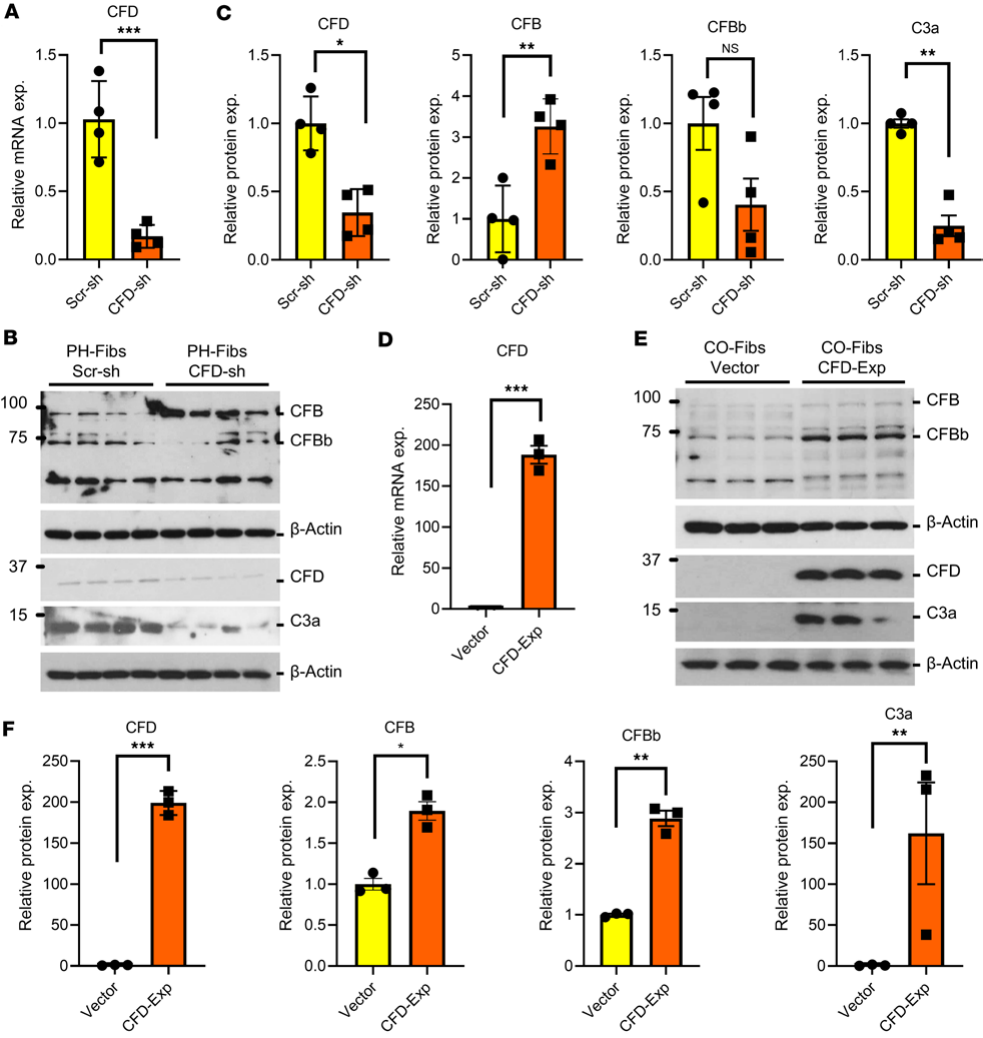
CFD regulates the activation of C3 in PA adventitial fibroblasts. (**A**) qRT-PCR data show the mRNA level of CFD in scrambled-shRNA (Scr-sh) and CFD-knockdown (CFD-sh) PH-Fibs. (**B**) Western blot and (**C**) corresponding quantitative data of immunoblots from scrambled-shRNA and CFD-knockdown PH-Fibs. C3a denotes the activated component of C3, and CFBb represents the active fragment of CFB. The suppression of CFD through shRNA led to decreased levels of active components C3a and CFBb. (**D**) qRT-PCR data shows the overexpression of CFD in CO-Fibs utilizing the pLV-CFD expression vector. (**E**) Western blot and (**F**) corresponding quantitative data of immunoblots from control vector– and pLV-CFD expression vector–transfected CO-Fibs. C3a and CFBb indicate activation of C3 and CFB on overexpression of CFD in CO-Fibs. Paired 2-tailed *t* test was used to compare 2 groups of samples. Data are presented as mean ± SEM from bovine control fibroblasts (CO-Fibs Vector and CO-Fibs CFD Exp), *n* = 3; and PH fibroblast (PH-Fibs Scr-sh and PH-Fibs CFD-sh), *n* = 4. *P ≤ 0.05, **P < 0.01, ***P < 0.001.

CFD is a serine protease and a key, rate-limiting factor in the alternative pathway (28). Based on prior observations in other cell types (29–31), we tested whether CFD in PH-Fibs acted intracellularly to generate active complement components. Using shRNA silencing, we generated bovine PH-Fibs with significantly decreased CFD mRNA levels compared with short hairpin–scrambled controls (Figure 4, A and B). This resulted in a robust knockdown of CFD at the protein level, leading to a corresponding decrease in CFBb and C3a (activated fragment of C3) levels in CFD-knockdown cells (Figure 4, B and C).

Conversely, overexpression of CFD in CO-Fibs led to a marked increase in CFBb and C3a levels (Figure 4, D–F). These observations collectively support the hypothesis that CFD acts intracellularly to activate C3. To complement these findings, we employed the pharmacological CFD inhibitor ALXN2050 (vemircopan) to target CFD activity (32). The CFD inhibitor treatment reduced CFB and C3 activation, leading to decreased C3a production in PH-Fibs. While the effect of the CFD inhibitor on CFB activation was not as pronounced as that of CFD knockdown, and while we did not observe a significant decrease in CFBb levels after the inhibitor treatment, total CFB levels increased, suggesting decreased CFB activation following ALXN2050 treatment (Supplemental Figure 2A). We also examined the effect of the CFD inhibitor on C3a in fibroblast conditioned media (serum-free) and detected a decrease in C3a in the media with CFD inhibitor treatment (Supplemental Figure 2B).

### CFD is localized in the cytoplasmic region of adventitial fibroblasts and interacts with and activates CFB.

**Figure 5:**
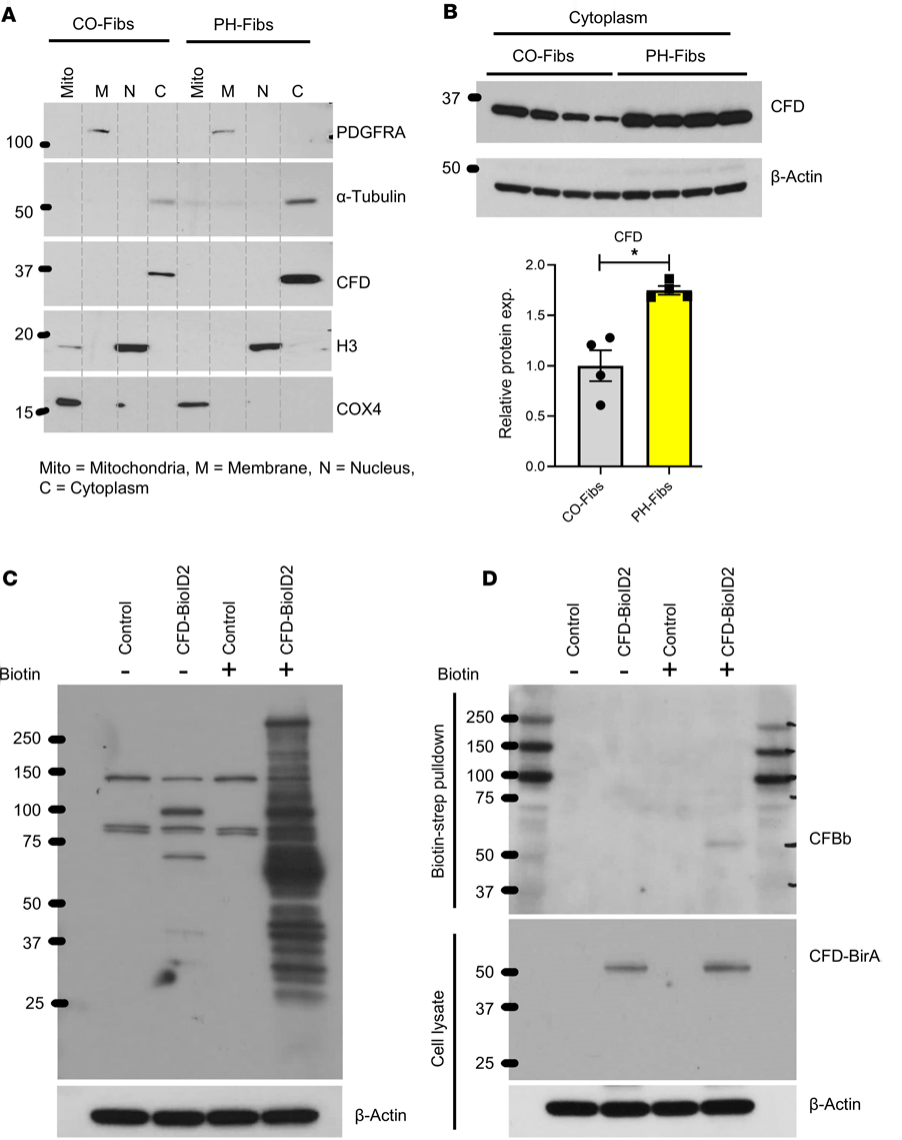
CFD is localized in the cytoplasmic region of adventitial fibroblasts and interacts with and activates CFB. (**A**) Immunoblot analysis was conducted to determine the subcellular localization of CFD in adventitial fibroblasts. CO-Fibs and PH-Fibs were fractionated into mitochondria (Mito), membrane (M), nucleus (N), and cytoplasm (C). The immunoblots were probed with antibodies against PDGFRA, α-tubulin, CFD, H3, and COX4. The results indicate that CFD is localized in the cytoplasm. (**B**) Immunoblotting and quantitative analysis of CFD in the cytoplasmic fractions from CO-Fibs and PH-Fibs revealed an increased level of CFD in PH-Fibs. (**C**) Biotinylation was assessed in IPAH fibroblasts transfected with control and CFD-BioID2 (CFD-BirA) constructs. Western blot analysis of total cell lysates shows the presence of biotinylated proteins after biotin treatment (50 μM). The biotinylated proteins were probed with streptavidin-HRP, and β-actin was used as a loading control. (**D**) Western blot analysis of streptavidin-based pulldown of biotinylated proteins from control and CFD-BioID2 plasmid–transfected IPAH fibroblasts both with and without biotin treatment was performed. The immunoblot was probed with antibodies against CFB, CFD, and β-actin. The proximity ligation assay indicates a potential interaction between CFD and CFB in adventitial fibroblasts. Unpaired 2-tailed *t* test was used to compare 2 groups of samples. Data are presented as mean ± SEM; *n* = 4. *P ≤ 0.05.

CFD expression in fibroblasts significantly influences the activation of CFB and C3, as demonstrated above. To further investigate how CFD promotes the activation of CFB and C3 in fibroblasts, we first examined localization of CFD in adventitial fibroblasts. Subcellular fractionation of CO-Fibs and PH-Fibs was performed to assess the localization of CFD across different subcellular compartments (nucleus, mitochondria, cell membrane, and cytoplasm). Our findings revealed that CFD was specifically localized in the cytoplasmic fraction, with significantly higher expression observed in PH-Fibs (Figure 5, A and B).

Next, we explored the potential interaction between CFD and CFB using a proximity ligation assay. Fibroblasts were transfected with the pLV-CFD-BioID2 vector and treated with biotin. Following this, biotinylated proteins were pulled down using streptavidin magnetic beads. The proximity ligation assay demonstrated that CFD interacted with CFB in cells, and activated fragments of CFB were enriched in the biotin-streptavidin pull-down (Figure 5, C and D).

These findings underscore the critical role of intracellular CFD and its interaction with CFB in facilitating the activation of C3 into C3a within adventitial fibroblasts.

### C3aR1 expression is localized on the cell membrane and mitochondria in pulmonary vascular fibroblasts.

**Figure 6:**
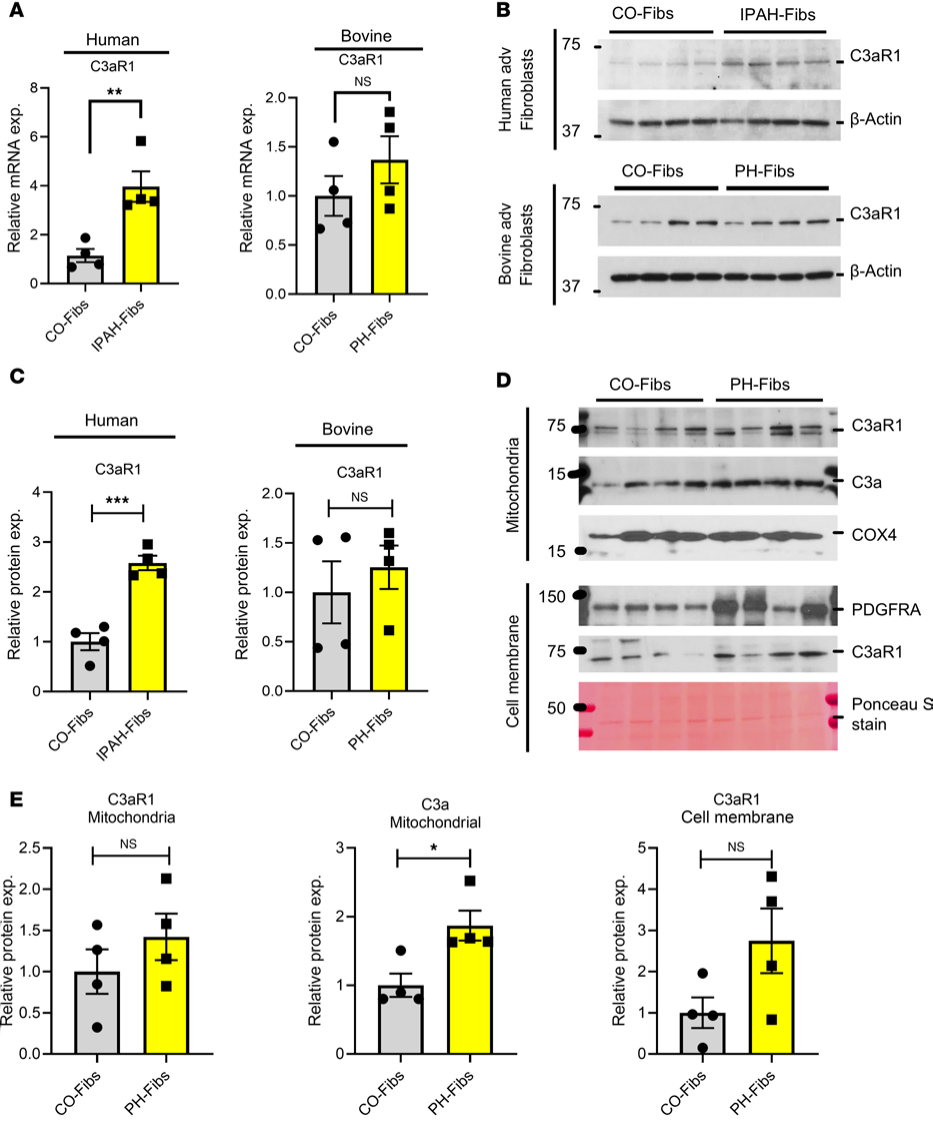
C3aR1 expression is localized on the cell membrane and mitochondria in pulmonary vascular fibroblasts. (**A**) qRT-PCR data representing mRNA expression of C3aR1 in human IPAH and bovine PH-Fibs compared with their respective control fibroblasts. (**B** and **C**) Western blot and quantitative data of Western blot of C3aR1 representing expression of C3aR1 in IPAH/PH-Fibs (human and bovine) and their related control fibroblasts. (**D** and **E**) Western blot analysis of C3aR1 from the isolated membrane and mitochondria of bovine PH-Fibs and CO-Fibs and corresponding quantitative immunoblot data. The cell membrane C3aR1 protein level was normalized using Ponceau S stain; mitochondrial C3aR1 protein was normalized using COX4; and PDGFRA protein used to conform the cell membrane fraction. Unpaired 2-tailed *t* test was used to compare 2 groups of samples. Data are presented as mean ± SEM from bovine CO- and PH-Fibs, *n* = 4; and human control and IPAH fibroblasts: *n* = 4. *P ≤ 0.05 **P < 0.01, ***P < 0.001.

Activation of cells can occur through the binding of C3a to cell-surface C3a receptors. Recent evidence also suggests that C3a binds to internal C3a receptors located on mitochondria and/or lysosomes (14, 33). To investigate this further, we examined C3aR1 expression in human IPAH-Fibs, bovine PH-Fibs, and their respective controls. Our analysis revealed increased C3aR1 expression, particularly in human cells, at both the RNA and protein levels (Figure 6, A–C).

We also explored the localization of C3aR1 within fibroblasts. Subcellular fractionation of control and PH fibroblasts was performed to determine the localization of C3aR1 across different subcellular compartments. Notably, we found C3aR1 expression to be localized on the cell membrane and mitochondria, with PH-Fibs exhibiting a trend toward higher levels of expression compared with CO-Fibs (Figure 6, D and E).

### CFD knockdown normalizes metabolic reprogramming in PH-Fibs.

**Figure 7:**
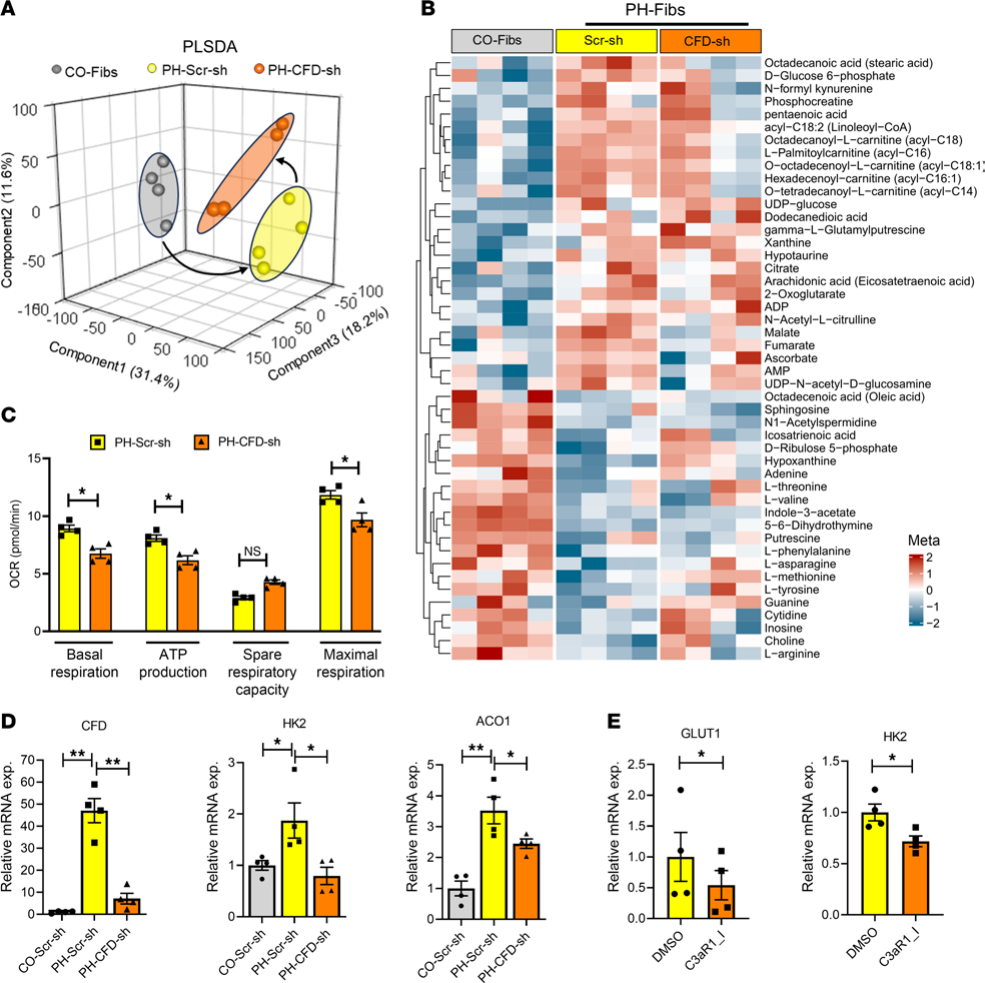
CFD knockdown reduced the altered metabolic reprogramming in bovine PH-Fibs. (**A**) Partial least squares discriminant analysis (PLS-DA) plot shows the separation of CO-Fibs and PH-Fibs (Scr-sh and CFD-sh) groups based on their metabolic profiles. (**B**) Heatmap representing altered metabolites in Scramble-transfected PH-Fibs compared with CO-Fibs and CFD-knockdown PH-Fibs. CFD knockdown notably affects metabolites associated with glycolysis, the TCA cycle, and fatty acid metabolism. (**C**) Mitochondrial respiration in PH-Scr-sh– and PH-CFD-sh–transfected fibroblasts. The results indicate a decrease in basal respiration, ATP production, and maximal respiration following CFD knockdown. However, spare respiratory capacity increased on CFD knockdown. (**D**) HK2 and ACO1 mRNA expression levels were analyzed in CO-Scr-sh, PH-Scr-sh, and PH-CFD-sh stably transfected fibroblasts. Knockdown of CFD led to a significant decrease in expression levels of HK2 and ACO1 in PH-Fibs. (**E**) Treatment with C3aR inhibitor (SB290157) at a concentration of 20 μM in IPAH fibroblasts led to a reduction in the levels of GLUT1 and HK2 mRNA. For comparisons involving more than 2 groups with one variable, 1-way ANOVA followed by Holm-Šidák post-hoc test was used; and a paired 2-tailed *t* test was used to compare 2 groups of samples. Data are presented as mean ± SEM from bovine CO-Fibs/CO-Fibs transfected with Scr-sh and PH-Fibs (Scr-sh and CFD-sh transfected), human IPAH fibroblasts (DMSO- and C3aR1 inhibitor treated); *n* = 4. *P ≤ 0.05, **P < 0.01.

Metabolic reprogramming of hypertensive adventitial fibroblasts in PH is well established, though the underlying mechanisms remain unclear (25). We sought to determine the role of CFD in fibroblast metabolism. To this end, we analyzed CO-Fibs and PH-Fibs treated with scrambled shRNA (Scr-sh) or CFD shRNA (CFD-sh) using ultra-high-pressure liquid chromatography/MS–based (UHPLC/MS-based) metabolomics analyses. Our data revealed that PH-Fibs exhibited enhanced activation of glycolysis, TCA cycle, and fatty acid metabolism pathways, which were reduced by CFD knockdown (Figure 7, A and B, and Supplemental Figure 4A).

Consistent with this finding, CFD knockdown in PH-Fibs led to decreased levels of specific metabolites associated with glycolysis (glucose-6-phosphate), the TCA cycle (fumarate and malate), and fatty acid metabolism (hexadecenoyl-carnitine, octadecanoic acid, and *O*-octadecenoyl-l-carnitine) (Supplemental Figure 3, A–C). Pathway enrichment analysis of 135 metabolites, performed using MetaboAnalyst and the Small Molecule Pathway Database (SMPDB), showed enrichment of mitochondrial fatty acid metabolism and the TCA cycle in PH-Fibs compared with the control group. However, CFD knockdown markedly reduced the enrichment of these pathways (Supplemental Figure 4B).

Further, mitochondrial function parameters were assessed using the Seahorse XFe96 Cell Mito Stress Test. Results demonstrated a decrease in basal respiration, maximal respiration, and ATP production–coupled respiration in CFD-knockdown PH-Fibs compared with Scr-sh–treated PH-Fibs (Figure 7C and Supplemental Figure 5, A and B). These observations underscore a reduction in metabolite levels and mitochondrial activity in PH-Fibs following CFD knockdown.

To investigate whether CFD knockdown influenced the expression of metabolic genes in PH-Fibs, we analyzed gene expression changes. Our findings revealed a significant decrease in expression of glycolysis-associated genes (GLUT1, HK2, GPI) and the TCA cycle gene (ACO1) in PH-Fibs with CFD knockdown (Figure 7D and Supplemental Figure 6A). In contrast, overexpression of CFD significantly increased the levels of GLUT1 and HK2 in PH-Fibs (Supplemental Figure 6B).

To further explore whether the effects of CFD were mediated through C3a, we treated CFD-knockdown PH-Fibs with a C3a peptide. Exogenous C3a treatment reversed the effects of CFD knockdown on GLUT1 and HK2 gene expression (Supplemental Figure 7, A–C). Additionally, we examined the effect of C3aR inhibition on metabolic gene expression. Treatment of IPAH fibroblasts with the small-molecule C3aR inhibitor SB290157 resulted in decreased expression of GLUT1 and HK2 in PH-Fibs, mirroring the phenotype observed with CFD knockdown (Figure 7E).

### CFD knockdown decreases expression of proinflammation genes in PH-Fibs.

**Figure 8:**
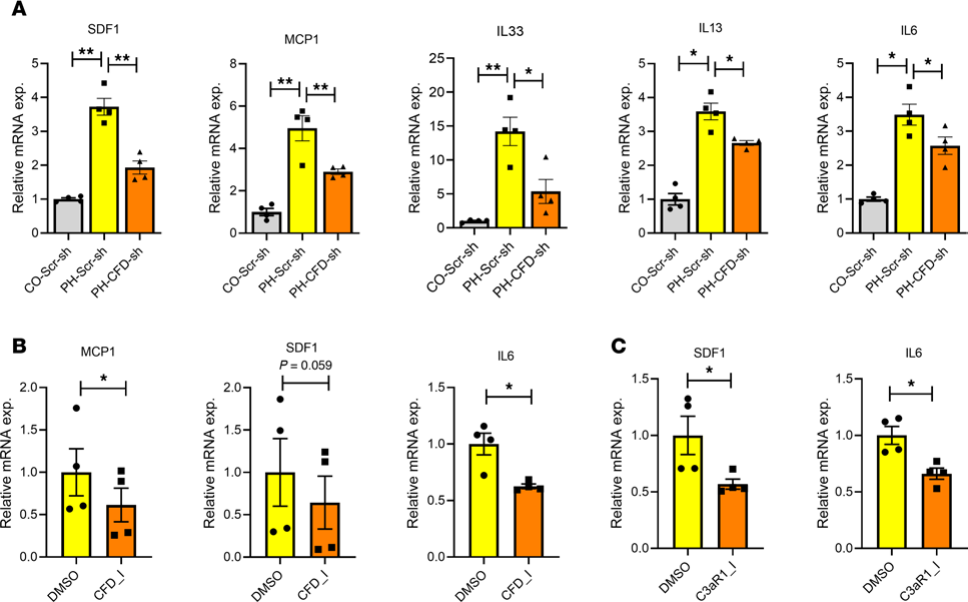
CFD knockdown decreased expression of proinflammatory genes in PH-Fibs. (**A**) qRT-PCR analysis of cytokines/chemokines in bovine control and PH-Fibs were stably transfected with either scramble or CFD shRNA. CFD knockdown in PH-Fibs reduced levels of proinflammatory genes, including SDF1, MCP1, IL-6, IL-33, and IL-13. (**B**) Pharmacological inhibition of CFD in PH-Fibs using ALXN2050 (vemircopan) at a concentration of 10 μM results in a reduction in expression levels of MCP1, SDF1, and IL-6. (**C**) C3aR inhibition using SB290157 at a concentration of 20 μM in IPAH fibroblasts results in a decrease in expression of SDF1 and IL-6. For comparisons involving more than 2 groups with one variable, 1-way ANOVA followed by Holm-Šidák post-hoc test was used; and paired 2-tailed *t* test was used to compare 2 groups of samples. Data are presented as mean ± SEM from bovine CO-Fibs (Scr-sh transfected) and PH-Fibs (Scr-sh and CFD-sh transfected), bovine PH-Fibs (DMSO and CFD inhibitor treated), and human IPAH fibroblasts (DMSO and C3aR1 inhibitor treated); *n* = 4. *P ≤ 0.05, **P < 0.01.

In recent years, inflammation has emerged as a pivotal area of interest, with the immune system increasingly recognized as a key player in the pathobiology of PH (4). Given the well-established association between metabolic abnormalities in vascular stromal cells and inflammatory gene expression, we investigated whether CFD knockdown would affect inflammatory gene expression in PH-Fibs. Our analysis revealed a marked increase in multiple inflammatory genes, including MCP1, SDF1, IL-6, IL-33, and IL-13 in PH-Fibs compared with CO-Fibs. Notably, these were significantly reduced in CFD-sh–treated PH-Fibs (Figure 8A). In contrast, overexpression of CFD significantly increased levels of inflammatory genes, including MCP1, SDF1, IL-6, and IL-13 (Supplemental Figure 8, A–D).

We further evaluated the effect of targeting CFD pharmacologically using ALXN2050 in PH-Fibs. Treatment with the CFD inhibitor significantly decreased expression of proinflammatory genes, including MCP1, SDF1, and IL-6 (Figure 8B). Additionally, we examined the effect of the C3aR1 inhibitor on inflammatory gene expression. The C3aR1 inhibitor significantly reduced expression levels of both SDF1 and IL-6 in IPAH-Fibs (Figure 8C).

## Discussion

The findings in this study underscore a critical and previously underexplored role for intracellular complement proteins (complosome), particularly C3, CFD, and CFB, in driving the metabolic and proinflammatory reprogramming of hypertensive PA fibroblasts. Through single-cell sequencing analysis of distal PAs from humans and young bovine calves with severe PH, we identified increased expression of C3, CFD, and CFB in fibroblasts isolated from PH subjects. Immunohistochemical staining further confirmed elevated CFD expression at the protein level in the adventitia of PAs from patients with IPAH. Complementing these findings, cultured cells from these vessels showed persistently elevated C3, CFD, and CFB mRNA expression, accompanied by a more open chromatin structure in these genes in PH-Fibs compared with controls.

Importantly, our data revealed enhanced activation of C3 and CFB in PH-Fibs, as indicated by increased levels of their activation products, C3a and CFBb, respectively, compared with CO-Fibs. We also demonstrated the presence of C3aR1 in adventitial fibroblasts, with significantly higher expression in IPAH-Fibs versus CO-Fibs. Subcellular fractionation confirmed the presence of C3aR1 on cell membranes and mitochondria, with further analysis revealing mitochondrial localization of C3a. Analysis of fibroblast-conditioned media also indicated the release of the activated C3 fragment C3a in the medium. Collectively, these results demonstrate the existence of an active complosome in PH adventitial fibroblasts, representing what we believe to be a new mechanism in the pathogenesis of PH.

We hypothesized that CFD is essential for activating C3 in fibroblasts. Our findings provide evidence that intracellular CFD activates C3, producing C3a, which engages C3aR1 on cell membranes and mitochondria. The findings clearly demonstrate that intracellular complement had a strong effect on metabolism and other aspects of the persistently activated fibroblast phenotype (inflammation, proliferation, apoptosis resistance) that have been well described (25). A major challenge involves discerning whether the complement produced by cells is acting solely intracellularly, functions at the cell surface after secretion, or both. Our results demonstrate that CFD was localized in cytoplasm and activated the C3 through interaction with CFB, leading to production of C3a. Further analysis shows that C3a was localized on mitochondria and also released from fibroblasts. This supports previously reported observations showing that the C3a functions intracellularly and locally (29, 34). Remarkably, CFD knockdown normalized the metabolic reprogramming observed in PH-Fibs, specifically reversing abnormalities in the TCA cycle, and fatty acid metabolism. Furthermore, CFD knockdown led to significant reductions in expression of metabolic and proinflammatory genes, including GLUT1, HK2, GPI, ACO1, IL-6, MCP1, SDF1, IL-33, and IL-13. These findings highlight CFD as a critical regulator of the activated phenotype of PH-Fibs, although the precise mechanisms through which CFD controls gene expression remain to be elucidated.

The findings of this study align with recent discoveries that certain cell types express complement proteins necessary for autocrine complement activation (35). Such pathways are pivotal for cellular homeostasis, activation, and inflammation (36, 37). For instance, cell-intrinsic C3 expression in synovial fibroblasts primes tissue sites for inflammation and is associated with enhanced aerobic glycolysis and increased expression of the glucose transporter GLUT1, driving a proinflammatory phenotype (13). Similarly, intracellular C3 stores in lung epithelial cells enhance cellular survival under hypoxic conditions and contribute to autocrine signaling during stress (19, 38).

Our findings also contribute to the understanding of the complosome’s role in cellular metabolism. Intracellular C3a and C5a — via interactions with mitochondrial, lysosomal, or endosomal C3a and C5a receptors — modulate core physiological processes, including autophagy, gene expression, and metabolism (17, 22, 34, 39). Notably, complosome activity in lysosomes and mitochondria, as hotspots of metabolic machinery, directly influences glycolysis, fatty acid metabolism, and oxidative phosphorylation (19, 40–43). This study reinforces the connection among the complosome, metabolic reprogramming, and cellular adaptation to stress.

This work highlights the crucial role of intracellular CFD in driving metabolic and proinflammatory changes in PH. While primarily based on in vitro data, we believe that these observations pave the way for in vivo studies to fully elucidate CFD’s contribution to disease progression. Despite this limitation, our findings suggest that therapies targeting CFD, including repurposing drugs such as vemircopan, hold significant promise for treating PH, marking a new and exciting avenue for future research.

## Methods

### Sex as a biological variable.

In this study, we used human adventitial fibroblasts from both male and female donors (44); bovine fibroblasts were derived only from males, as females are not available for study (45–47).

### Animals.

The neonatal calf model of severe, chronic hypoxia-induced PH has been previously documented (45–47). In brief, 1-day-old male Holstein calves were exposed to hypobaric hypoxia (barometric pressure [PB]: 430 mmHg, 15,000 feet [4,570 m]) for 2 weeks (*n* = 4), while age-matched controls (*n* = 4) were kept at ambient altitude (PB: 640 mmHg, equivalent to 5,000 feet [1,520 m]). Standard veterinary care was administered in accordance with institutional guidelines at the Department of Physiology, School of Veterinary Medicine, Colorado State University. Animals were euthanized via an overdose of sodium pentobarbital (160 mg/kg body weight).

### Immunohistochemistry.

Immunohistochemical staining was conducted to detect expression of CFD in both human control and IPAH lung tissue, following the protocol described by Tuder et al. (5). Antigen retrieval was performed using citrate solution from Vector Laboratories. Formalin-fixed, paraffin-embedded (FFPE) lung tissue sections were stained with the primary CFD antibody (MyBioSource; MBS9203258). Visualization of antibody binding was achieved using the Dako polymer-labeled, HRP-bound secondary reagent (Dako EnVision^+^ HRP rabbit). Light green was used as the counterstain.

### Cell culture.

PA samples were collected by the Division of Thoracic Surgery, Medical University of Vienna, from patients with IPAH (*n* = 4) and healthy donors (*n* = 4) as a control, and adventitial fibroblasts were precisely isolated. The demographics of IPAH patients and donors and cell characterizations have been previously described by our research group (44). Bovine adventitial fibroblasts were isolated with equal precision from the PA of 2-week-old male calves with severe PH and age-matched controls. Isolated adventitial explants from the PA were cultured as previously described (26, 48). Both human and bovine adventitial fibroblasts were cultured in DMEM media supplemented with 4 mM glutamine (Gibco), 25 mM HEPES, 1 mM pyruvate (Gibco), penicillin-streptomycin 1× (Gibco), and 10% bovine calf serum (BCS). For the complement study, fibroblasts were cultured in serum-free DMEM media (supplemented with 4 mM glutamine, 25 mM HEPES, 1 mM pyruvate, and penicillin-streptomycin 1×) for 48 hours. All cells were maintained in a 5% CO_2_ humidified environment at 37°C. Experiments were conducted on cells within passages 3–6. PH/IPAH fibroblasts were treated with the CFD inhibitor ALXN2050 (vemircopan; HY-139588, MedChemExpress) at a concentration of 10 μM and the C3aR inhibitor (SB290157; HY-101502A, MedChemExpress) at a concentration of 20 μM for 24 hours.

### Real-time reverse transcriptase–polymerase chain reaction.

Total fibroblast RNA was extracted (NucleoSpin, 740955.250) and reverse-transcribed to cDNA (Bio-Rad, 1708891). All SYBR primer sets for real-time reverse transcriptase–polymerase chain reaction (qRT-PCR) are listed in Supplemental Table 1. The qRT-PCR experiment was run on a QuantStudio 6 Flex System (Applied Biosystems), and data were analyzed on QuantStudio RT-PCR software. Results were normalized to HPRT using the relative quantitative ΔΔCt method, and fold change in expression was calculated relative to the control in each group.

### Western blotting.

Primary adventitial fibroblasts were serum starved for 48 hours and were lysed using RIPA buffer (Thermo Fisher Scientific, 89900) supplemented with a protease inhibitor cocktail (Thermo Fisher Scientific, 78438). The cell lysate was centrifuged at 16,000*g* for 15 minutes at 4°C to remove cellular debris. The protein concentration of isolated proteins was determined using the bicinchoninic acid (BCA) method. Subsequently, 30–50 μg protein was separated by electrophoresis on 4%–20% Bis-Tris SDS-PAGE and transferred to a PVDF membrane (Millipore) using a wet transfer system. The membrane was blocked with 5% skim milk and then incubated with one of the following primary antibodies — anti-C3, 1:5,000 (ABclonal, A16781), anti-C3a, 1:50 (Hycult Biotech; HM207), anti-CFB, 1:3,000 (ABclonal, A1706), anti-CFD, 1:3,000 (MyBioSource, MBS9203258; Thermo Fisher Scientific, PA5-79034), anti-C3aR1, 1:1,000 (Santa Cruz Biotechnology Inc., sc-133172), anti-H3, 1:2,000 (Cell Signaling Technology, 9715S), anti–α-tubulin, 1:5,000 (MilliporeSigma, T6199), anti-PDGFRA, 1:3,000 (Thermo Fisher Scientific, PA534739), and anti–β-Actin, 1:10,000 (MilliporeSigma, A5316) — overnight at 4°C. This was followed by incubation with one of the HRP-conjugated secondary antibodies: anti-rabbit, 1:20,000 (Invitrogen, 31480) and anti-mouse, 1:20,000 (Invitrogen, 31430) for 1 hour at room temperature. The protein bands were visualized using chemiluminescence (MilliporeSigma) and captured on x-ray film. β-Actin was utilized as an internal loading control.

### Plasmids and transfection.

CFD-specific shRNA, scrambled control, CFD-overexpression, and CFD-BioID2 plasmids were synthesized by Vector Builder. Sequences are provided in Supplemental Table 2. Lentiviral particles were generated by cotransfecting shRNA/CFD-Expression/CFD-BioID2 vectors with a lentiviral packaging mix (MilliporeSigma, SHP001) in 293T cells using Lipofectamine 3000 (Invitrogen, L3000008). Lentiviral supernatant was filtered (4 μm), supplemented with Polybrene (10 μg/mL) (Santa Cruz Biotechnology Inc., sc-134220), and applied to CO/PH-Fibs for 24 hours. Stably transfected cells and positive control were selected with puromycin (3 μg/mL) (InvivoGen, ant-pr-1) for 72 hours.

### Subcellular fractionation.

Bovine fibroblasts (CO-Fibs, *n* = 4, and PH-Fibs, *n* = 4) were rinsed with PBS and then scraped into cold PBS. The cell suspension was centrifuged at 400*g* for 10 minutes at 4°C to collect the cell pellets. The pellets were resuspended in ice-cold mitochondrial isolation buffer (20 mM HEPES, 10 mM KCl, 2 mM MgCl_2_, 1 mM EGTA, 1 mM EDTA, 1 mM DTT, and 1× protease inhibitor) and homogenized using a Dounce homogenizer. The homogenates were centrifuged at 1,000*g* for 10 minutes at 4°C to isolate the nuclear fraction. The nuclear pellets were further processed by passing them through a 25-gauge needle 10 times, followed by another centrifugation at 1,000*g* for 10 minutes at 4°C to obtain purified nuclei. The supernatants from the initial centrifugation step were further centrifuged at 14,000*g* for 15 minutes at 4°C to collect the mitochondrial fraction. This process was repeated with the supernatants to ensure recovery of any remaining mitochondria. Subsequently, the supernatants from the mitochondrial isolation step were ultracentrifuged at 100,000*g* for 1 hour. The resulting pellets represented the plasma membrane fraction, while the supernatants contained the cytoplasmic fraction. Nuclear, mitochondrial, and membrane fractions were resuspended in RIPA buffer for protein extraction.

### Proximity ligation assay.

The pLV-CFD-BioID2 vector was stably transfected in IPAH fibroblasts. 4 × 10^7^ fibroblasts (control and CFD-BioID2–transfected) were incubated with 50 μM biotin for 16 hours. Cells were washed with PBS and lysed with 1 mL lysis buffer containing 25 mM Tris-HCl pH 7.6, 150 mM NaCl, 1% NP-40, 1% sodium deoxycholate, 0.1% SDS, and 1× complete protease inhibitor. After lysis, lysates were centrifuged at 16,500*g* for 15 minutes. The supernatant was collected in a 1.5 mL tube and incubated with 300 μL Dynabeads (Thermo Fisher Scientific, 65001) overnight at 4°C. The next day, Dynabeads were collected using a magnetic stand and washed twice with 2% (wt/vol) SDS and twice with lysis buffer. Beads were resuspended in Laemmli buffer containing 2 mM biotin. Equal amounts of control and CFD-BioID2 protein samples, with or without biotin treatment, were loaded on SDS-PAGE and transferred to the PVDF membrane. Pulldown protein samples were probed with CFD and CFB antibodies to detect the possible interaction between CFD and CFB in fibroblasts.

### UHPLC/MS-based metabolomics analyses.

Metabolomics analyses were performed as previously described (49–51), with minor modifications. Briefly, CO-Fibs and PH-Fibs (Scr-sh and CFD-sh) (*n* = 4 each group) were cultured in serum-free DMEM. After rinsing and scraping in ice-cold PBS, cells were pelleted. For analysis, cell lysis was performed using ice-cold buffer (methanol/acetonitrile/water, 5:3:2, vol/vol/vol) (2 × 10^6^ cells/mL buffer). The analytical platform employs a 5-minute C18 gradient on a Vanquish UHPLC system coupled to a Q Exactive mass spectrometer (Thermo Fisher Scientific) in positive and negative ion modes (separate runs) (52). Metabolomics data were analyzed using MetaboAnalyst (version 6.0). The data were normalized by probabilistic quotient normalization (PQN). Subsequent transformation and scaling were performed using square root transformation and Pareto scaling. Various statistical methods, including 1-way ANOVA and multivariate analysis (PLS-DA), were employed to identify changes in metabolomics data between CO-Fibs, PH-Fibs-Scr-sh, and PH-Fibs-CFD-sh.

### Seahorse metabolic flux measurements.

Seahorse XFe Cell Mito Stress Tests were conducted following the manufacturer’s protocol (Agilent, C103015-100) on an XFe 96 instrument. PH-Scr-sh and PH-CFD-sh fibroblasts were cultured in a Seahorse 96-well plate. One day before the assay, the sensor cartridge was hydrated in sterile tissue culture-grade water overnight at 37°C in a non-CO_2_ incubator. The next day, the sensor cartridge was incubated in XF calibrant buffer for 3 hours. On the day of the assay, PH-Scr-sh and PH-CFD-sh fibroblasts were incubated with the Cell Mito Stress assay medium, which was supplemented with 10 mM glucose, 1 mM pyruvate, and 2 mM glutamine for 1 hour prior to the experiment. The first measurement taken was the basal respiration for PH-Scr-sh and PH-CFD-sh fibroblasts. Subsequently, ATP production was inhibited using oligomycin (0.75 μM), followed by addition of FCCP (2 μM). Finally, the electron transport chain was inhibited using rotenone/antimycin (0.5 μM). The mitochondrial oxygen consumption rate (OCR) data obtained from the Seahorse metabolic flux analysis were normalized to protein concentration.

### scRNA-Seq analysis.

Distal PAs were dissected from the right caudal lung lobe of 3 control calves and 3 calves with PH following a 14-day hypoxia exposure protocol, as previously described (24, 53). Briefly, a single-cell suspension was prepared from dPAs and suspended in PBS containing 0.04% BSA. The cells were introduced into microfluidic channels designed to capture single cells for downstream analysis. The captured cells were lysed to release RNA, which was then barcoded through reverse transcription within individual gel bead-in emulsions (GEMs). Reverse transcription was performed on an S1000TM Touch Thermal Cycler (Bio-Rad) at 53°C for 45 minutes, followed by 85°C for 5 minutes, and then held at 4°C. The resulting cDNA was amplified, and its quality was assessed using an Agilent 4200 system. scRNA-Seq libraries were constructed following the manufacturer’s instructions using the Single Cell 3′ Library and Gel Bead Kit V3.1 (10x Genomics) (54). The libraries were sequenced on an Illumina NovaSeq 6000 sequencer with a sequencing depth of at least 100,000 reads per cell using the paired-end 150 bp strategy.

Sequence reads were aligned to the *Bos taurus* ARS-UCD1.2 reference genome using Cell Ranger v5 (10x Genomics). Raw read counts from all time points were aggregated and subjected to downstream analysis using Seurat (v4.3.0) (55). Low-quality cells, doublets, and damaged cells (defined as having >20% mitochondrial content) were excluded from the dataset using Seurat and DoubletFinder (v2.0.3) (56). After extensive bioinformatic quality control, sequencing data from 20,912 cells across all control dPAs and 15,692 cells across all PH dPAs were included in the analysis. The identified cell types in both control and PH dPAs, classified using the Bioconductor R packages “Single R” and “CellDex,” included structural cells (SMCs, fibroblasts, and ECs), as well as immune cells (macrophages, glia, dendritic cells, T cells, NK cells, mast cells, and neutrophils). This study primarily focused on fibroblasts, with EC and SMC populations used for comparative analysis. The cluster analysis represented as uniform manifold approximation and projection (UMAP) of bovine and human scRNA-Seq data (Figure 1) included human stromal cells: ECs *n* = 1,753, SMCs *n* = 3,126, and Fibs *n* = 4,523; bovine stromal cells included ECs *n* = 6,582, SMCs *n* = 10,901, and Fibs *n* = 1,016. Gene expression was visualized using dot plots produced with ggplot2 (v3.4.3), and heatmaps for average gene expression were created using the ComplexHeatmap (v2.14.0) library in R (v4.2.2). Human scRNA-Seq data were processed following the methodology outlined previously (23). This processing included steps such as quality control, normalization, clustering, and identification of cell types, ensuring accurate and reliable gene expression analysis at the single-cell level.

### Statistics.

The data are presented as mean ± SEM. Statistical analyses were performed using Prism 8.0 (GraphPad Software Inc.). An unpaired/paired 2-tailed *t* test was used to compare 2 groups of samples. For comparisons involving more than 2 groups with one variable, a 1-way ANOVA followed by a Holm-Šidák post-hoc test was conducted. Differences with *P* values of 0.05 or less were considered statistically significant.

### Study approval.

The lung explant specimens, provided by the Pulmonary Hypertension Breakthrough Initiative (PHBI), were processed following a standardized and systematic protocol. The use of samples from the PHBI was approved by the University of Colorado Institutional Review Board (COMIRB 08-0423). All procedures adhered to the *Guide for the Care and Use of Laboratory Animals* (National Academies Press, 2011) and were approved by the IACUCs of the University of Colorado Anschutz Medical Campus and the Department of Physiology, School of Veterinary Medicine, Colorado State University.

### Data availability.

All data point values in the graphs are reported in Supporting Data Values. The datasets used in this study are available in online repositories. The bovine scRNA-Seq data have been deposited in the NCBI’s Gene Expression Omnibus database (GEO GSE234156). The human scRNA-Seq data have been deposited as GEO GSE210248. The scRNA-Seq analysis codes are available from the authors upon request.

## Author contributions

RRP conceived the study, designed and performed the experiments, statistically analyzed and interpreted the data, created the figures, and wrote the manuscript. SK designed and performed the experiments, and statistically analyzed and interpreted the data. HZ and ML performed the experiments and edited the manuscript. CJH analyzed the data and edited the manuscript. SR and BAM performed the experiments. MGF designed the study. RMT performed the experiments, analyzed the data, and edited the manuscript. SC, GK, and KH provided support with scRNA-Seq data from human arteries and human primary fibroblasts, and edited the manuscript. KRS provided intellectual oversight of the project, study design, data interpretation, wrote and edited the manuscript.

## Supplementary Material

Supplemental data

Unedited blot and gel images

Supporting data values

## Version 1 02/13/2025

In-Press Preview

## Version 2 03/24/2025

Electronic publication

## References

[1] Humbert M (2019). Pathology and pathobiology of pulmonary hypertension: state of the art and research perspectives. Eur Respir J.

[2] Guignabert C (2024). Pathology and pathobiology of pulmonary hypertension: current insights and future directions. Eur Respir J.

[3] Jia Z (2023). Pulmonary vascular remodeling in pulmonary hypertension. J Pers Med.

[4] Stacher E (2012). Modern age pathology of pulmonary arterial hypertension. Am J Respir Crit Care Med.

[5] Tuder RM (2024). Digital spatial profiling identifies distinct molecular signatures of vascular lesions in pulmonary arterial hypertension. Am J Respir Crit Care Med.

[6] Frid MG (2020). Immunoglobulin-driven complement activation regulates proinflammatory remodeling in pulmonary hypertension. Am J Respir Crit Care Med.

[7] Ricklin D (2016). Complement in disease: a defence system turning offensive. Nat Rev Nephrol.

[8] Merle NS (2015). Complement system part I - molecular mechanisms of activation and regulation. Front Immunol.

[9] Paiano J (2019). Follicular B2 cell activation and class switch recombination depend on autocrine C3ar1/C5ar1 signaling in B2 cells. J Immunol.

[10] Liszewski MK (2013). Intracellular complement activation sustains T cell homeostasis and mediates effector differentiation. Immunity.

[11] Kolev M (2020). Diapedesis-induced integrin signaling via LFA-1 facilitates tissue immunity by inducing intrinsic complement C3 expression in immune cells. Immunity.

[12] Chaudhary N (2022). A single-cell lung atlas of complement genes identifies the mesothelium and epithelium as prominent sources of extrahepatic complement proteins. Mucosal Immunol.

[13] Friščić J (2021). The complement system drives local inflammatory tissue priming by metabolic reprogramming of synovial fibroblasts. Immunity.

[14] Ishii M (2021). Mitochondrial C3a receptor activation in oxidatively stressed epithelial cells reduces mitochondrial respiration and metabolism. Front Immunol.

[15] Mahajan S (2021). Local complement factor H protects kidney endothelial cell structure and function. Kidney Int.

[16] Tam JCH (2014). Intracellular sensing of complement C3 activates cell autonomous immunity. Science.

[17] Kolev M (2015). Complement regulates nutrient influx and metabolic reprogramming during Th1 cell responses. Immunity.

[18] Perucha E (2019). The cholesterol biosynthesis pathway regulates IL-10 expression in human Th1 cells. Nat Commun.

[19] Liszewski MK (2017). Complement’s hidden arsenal: new insights and novel functions inside the cell. Mol Immunol.

[20] Banda NK (2022). Analysis of complement gene expression, clinical associations, and biodistribution of complement proteins in the synovium of early rheumatoid arthritis patients reveals unique pathophysiologic features. J Immunol.

[21] Daugan MV (2021). Intracellular factor H drives tumor progression independently of the complement cascade. Cancer Immunol Res.

[22] King BC (2019). Intracellular cytosolic complement component C3 regulates cytoprotective autophagy in pancreatic beta cells by interaction with ATG16L1. Autophagy.

[23] Crnkovic S (2022). Single-cell transcriptomics reveals skewed cellular communication and phenotypic shift in pulmonary artery remodeling. JCI Insight.

[24] Plecitá-Hlavatá L (2023). Microenvironmental regulation of T-cells in pulmonary hypertension. Front Immunol.

[25] Zhang H (2024). Fibroblasts in pulmonary hypertension: roles and molecular mechanisms. Cells.

[26] Li M (2011). Emergence of fibroblasts with a proinflammatory epigenetically altered phenotype in severe hypoxic pulmonary hypertension. J Immunol.

[27] Wang D (2014). MicroRNA-124 controls the proliferative, migratory, and inflammatory phenotype of pulmonary vascular fibroblasts. Circ Res.

[28] Wiles JA (2018). Discovery and development of the oral complement factor D inhibitor danicopan (ACH-4471). Curr Med Chem.

[29] King BC, Blom AM (2024). Intracellular complement and immunometabolism: the advantages of compartmentalization. Eur J Immunol.

[30] O’Brien RM (2023). Thinking inside the box: intracellular roles for complement system proteins come into focus. Br J Cancer.

[31] Barratt J, Weitz I (2021). Complement factor D as a strategic target for regulating the alternative complement pathway. Front Immunol.

[32] Gavriilaki E (2022). Novel insights into factor D inhibition. Int J Mol Sci.

[33] Settembre C (2012). A lysosome-to-nucleus signalling mechanism senses and regulates the lysosome via mTOR and TFEB. EMBO J.

[34] West EE, Kemper C (2023). Complosome - the intracellular complement system. Nat Rev Nephrol.

[35] King BC, Blom AM (2023). Intracellular complement: evidence, definitions, controversies, and solutions. Immunol Rev.

[36] Strainic MG (2008). Locally produced complement fragments C5a and C3a provide both costimulatory and survival signals to naive CD4+ T cells. Immunity.

[37] Choy LN (1992). Adipsin and an endogenous pathway of complement from adipose cells. J Biol Chem.

[38] Kulkarni HS (2019). Intracellular C3 protects human airway epithelial cells from stress-associated cell death. Am J Respir Cell Mol Biol.

[39] Niyonzima N (2021). Mitochondrial C5aR1 activity in macrophages controls IL-1β production underlying sterile inflammation. Sci Immunol.

[40] Kolev M (2014). Complement--tapping into new sites and effector systems. Nat Rev Immunol.

[41] Arbore G (2018). Complement receptor CD46 co-stimulates optimal human CD8+ T cell effector function via fatty acid metabolism. Nat Commun.

[42] Kolev M, Kemper C (2017). Keeping it all going-complement meets metabolism. Front Immunol.

[43] King BC, Blom AM (2021). Complement in metabolic disease: metaflammation and a two-edged sword. Semin Immunopathol.

[45] Stenmark KR (1987). Severe pulmonary hypertension and arterial adventitial changes in newborn calves at 4,300 m. J Appl Physiol (1985).

[46] Applegate TJ (2021). Brief report: case comparison of therapy with the histone deacetylase inhibitor vorinostat in a neonatal calf model of pulmonary hypertension. Front Physiol.

[47] Li M (2021). Microenvironmental regulation of macrophage transcriptomic and metabolomic profiles in pulmonary hypertension. Front Immunol.

[48] El Kasmi KC (2014). Adventitial fibroblasts induce a distinct proinflammatory/profibrotic macrophage phenotype in pulmonary hypertension. J Immunol.

[49] D’Alessandro A (2015). Routine storage of red blood cell (RBC) units in additive solution-3: a comprehensive investigation of the RBC metabolome. Transfusion.

[50] Li M (2016). Metabolic reprogramming regulates the proliferative and inflammatory phenotype of adventitial fibroblasts in pulmonary hypertension through the transcriptional corepressor C-terminal binding protein-1. Circulation.

[51] Zhang H (2017). Metabolic and proliferative state of vascular adventitial fibroblasts in pulmonary hypertension is regulated through a MicroRNA-124/PTBP1 (polypyrimidine tract binding protein 1)/pyruvate kinase muscle axis. Circulation.

[52] Nemkov T (2019). High-throughput metabolomics: isocratic and gradient mass spectrometry-based methods. Methods Mol Biol.

[53] Frid MG (2009). Sustained hypoxia leads to the emergence of cells with enhanced growth, migratory, and promitogenic potentials within the distal pulmonary artery wall. Am J Physiol Lung Cell Mol Physiol.

[54] Kumar S (2024). Single cell transcriptomic analyses reveal diverse and dynamic changes of distinct populations of lung interstitial macrophages in hypoxia-induced pulmonary hypertension. Front Immunol.

[55] Hao Y (2021). Integrated analysis of multimodal single-cell data. Cell.

[56] McGinnis CS (2019). DoubletFinder: doublet detection in single-cell RNA sequencing data using artificial nearest neighbors. Cell Syst.

