# Supplementary material for: An intracellular complement system drives metabolic and proinflammatory reprogramming of vascular fibroblasts in pulmonary hypertension
Source: JCI Insight. 2025 Feb 13;10(6):e184141. doi: 10.1172/jci.insight.184141 (PMC11949053; doi:10.1172/jci.insight.184141)
Supplement: Supplemental data [file jciinsight-10-184141-s252.pdf]

An intracellular complement system drives metabolic and proinflammatory reprogramming of vascular fibroblasts in pulmonary hypertension

Ram Raj Prasad[1], Sushil Kumar[1], Hui Zhang[1], Min Li[1], Cheng-Jun Hu[2], Suzette Riddle[1], Brittany A McKeon[1], M. G. Frid[1], Konrad Hoetzenecker[3], Slaven Crnkovic[4,5], Grazyna Kwapiszewska[4,5], Rubin M Tuder[1,6], Kurt R Stenmark[1]

1. Cardiovascular And Pulmonary Research Laboratory (CVP), Department of Pediatrics and Medicine, University of Colorado, Anschutz Medical Campus
2. Department of Craniofacial Biology, University of Colorado, Anschutz Medical Campus
3. Department of Thoracic Surgery, Medical University of Vienna, Vienna, Austria
4. Ludwig Boltzmann Institute for Lung Vascular Research, Otto Loewi Research Center, Lung Research Cluster, Medical University of Graz, Graz, Austria.
5. Institute for Lung Health, Cardiopulmonary Institute, Member of the German Center for Lung Research, Justus Liebig University Giessen, Germany
6. Department of Lung Biology, University of Colorado, Anschutz Medical Campus

Corresponding author:

Kurt R Stenmark

CVP, Department of Pediatrics and Medicine, School of Medicine, RC2, Room N. 6124, University of Colorado Anschutz Medical Campus, Aurora, Colorado, USA 80045

Email: kurt.stenmark@cuanschutz.edu, Telephone: (303)724-5623

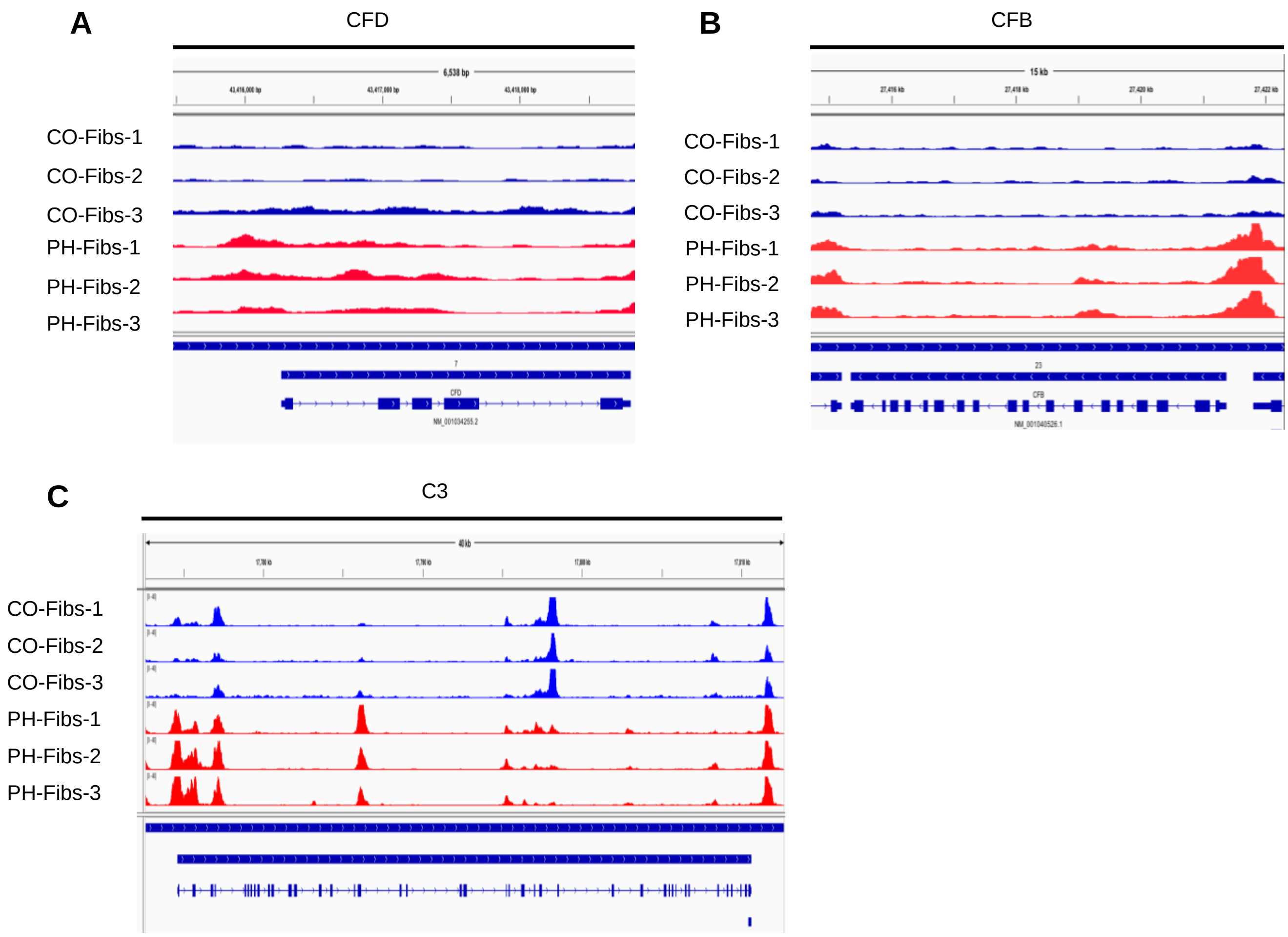

Supplementary Figure 1. Chromatin structure of CFD, CFB, and C3 genes is more open in PH-Fibs than in control fibroblasts.

A, B, and C) Assay for Transposase-Accessible Chromatin (ATAC) sequencing data reveals the open chromatin structure of CFD, CFB, and C3 genes in control fibroblasts (CO) and pulmonary hypertension fibroblasts (PH-Fibs). The results indicate that PH-Fibs exhibit higher levels of chromatin activation for CFD, CFB, and C3 compared to control fibroblasts.

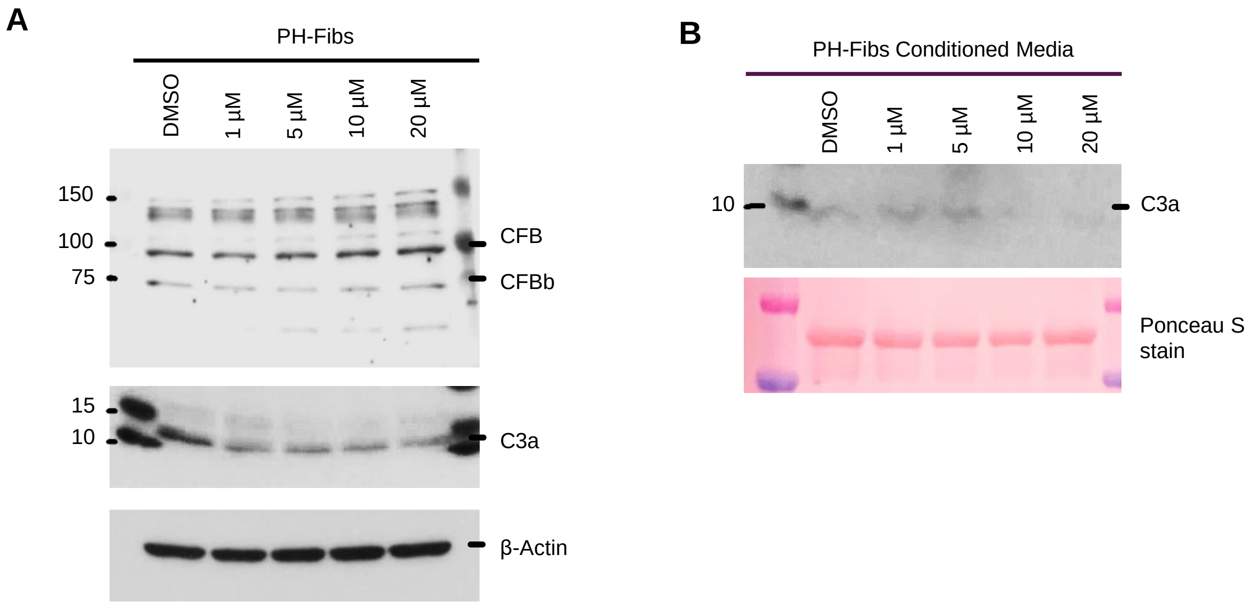

Supplementary Figure 2: CFD inhibitor (Vemircopan) treatment reduced the activation of CFB and C3 (C3a) in PH-Fibs. A) Vemircopan was tested at various concentrations (1-20 µM) to evaluate its inhibitory effect on CFD activity in PH-Fibs. The results indicate that Vemircopan effectively reduces the activation of C3 (C3a) and CFB. B) PH-Fibs condition media (serum-free) was collected after treatment with different concentrations of CFD inhibitor. Collected conditioned media was concentrated using 3kd cutoff protein concentrators (cat n. 88514), C3a level in conditioned media was assayed by immunoblotting, and Ponceau S stain was used as the loading control.

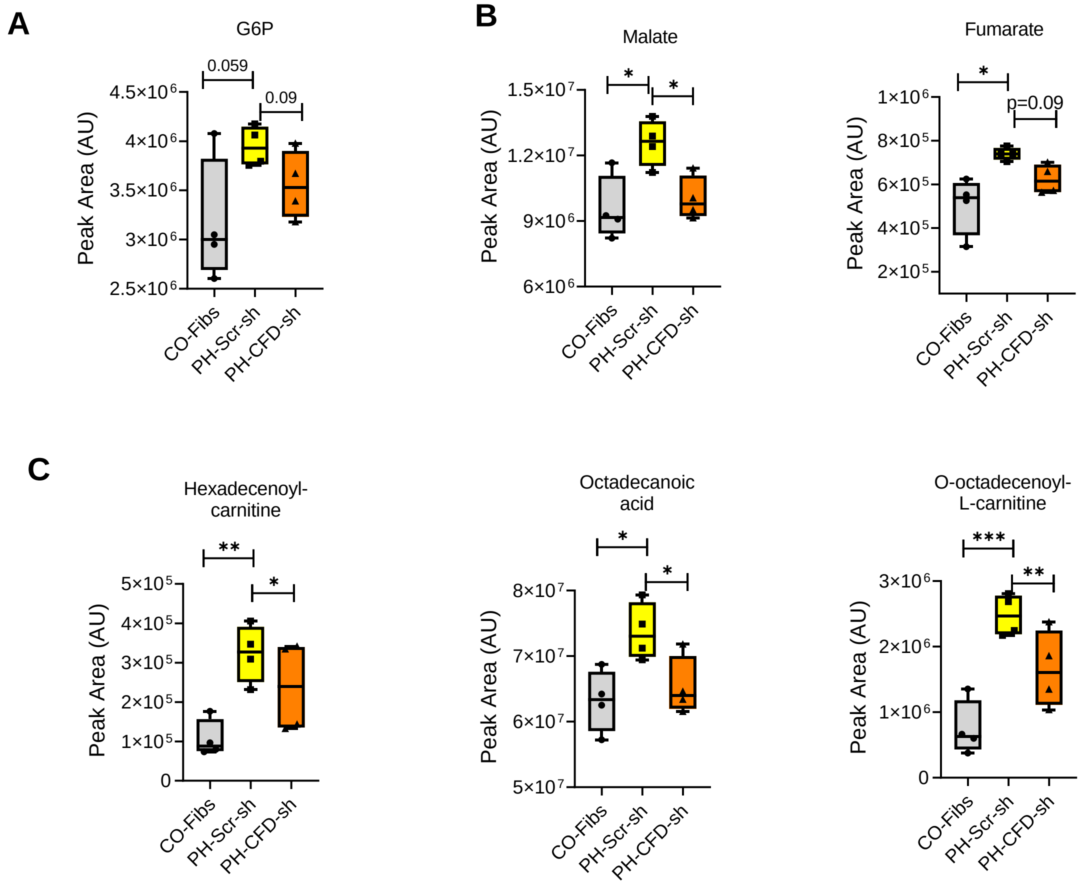

Supplementary Figure 3: CFD knockdown reduced the altered metabolites in PH-Fibs. A) The levels of glycolysis-related metabolites, specifically glucose-6-phosphate, were measured in PH-Fibs following the knockdown of CFD. B) Additionally, there was a significant decrease in the levels of TCA cycle metabolites, malate and fumarate, in PH-Fibs after CFD knockdown. C) Lastly, metabolites associated with fatty acid metabolism, including hexadecenoyl-carnitine (acyl-C16:1), O-octadecenoyl-L-carnitine (acyl-C18:1), and octadecanoic acid, also showed a significant reduction in PH-Fibs with CFD knockdown. For comparisons involving more than two groups with one variable, a one-way ANOVA followed by a Holm-Sidak post-test was used. Data are presented as mean ± SEM from control and PH fibroblast (Scr-sh and CFD-sh): n = 4, p-value ≤ 0.05 indicates a significant change. *P ≤0.05, **P < 0.01. ***P < 0.001.

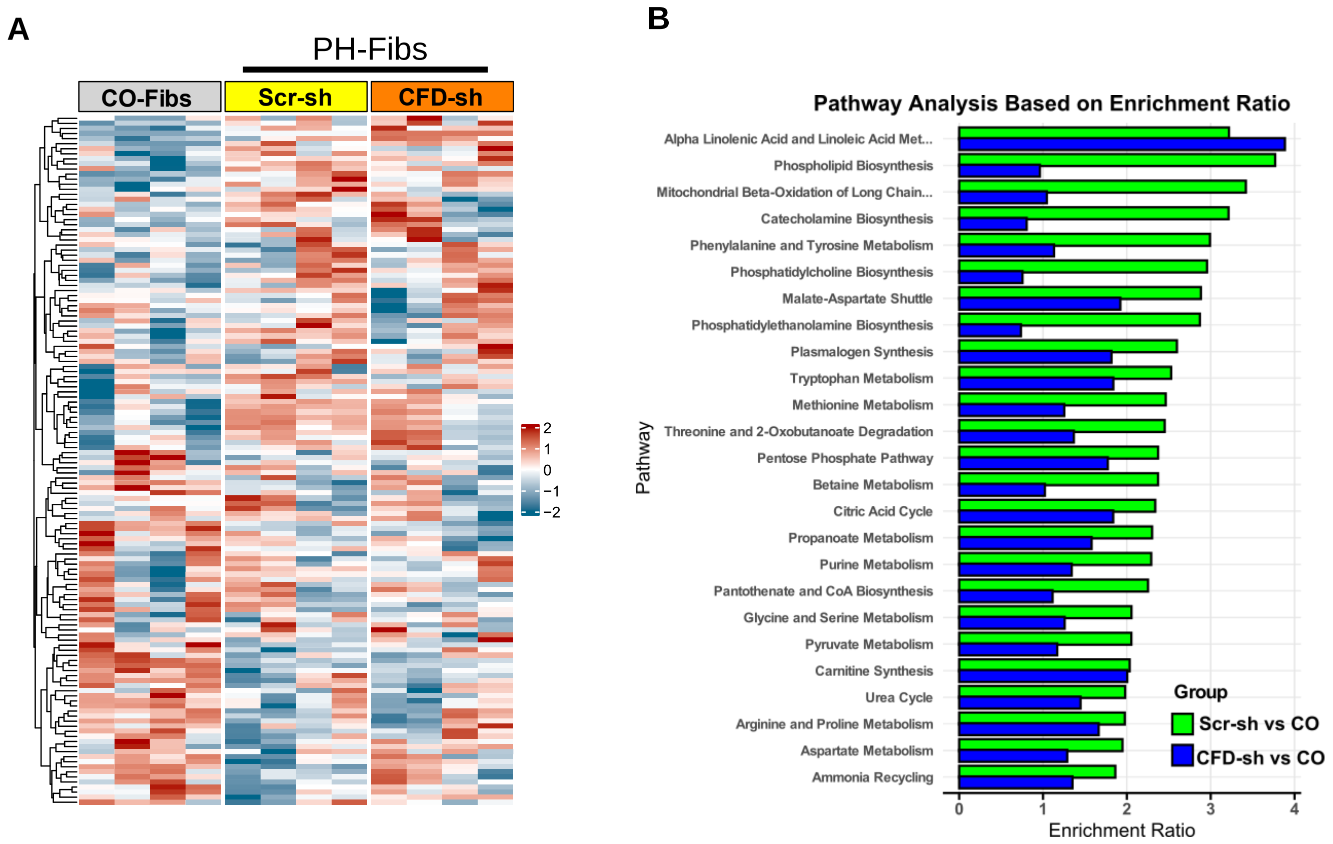

Supplementary Figure 4. Metabolic profiles and enrichment pathways analysis of CO-Fibs and PH-Fibs (treated with Scr-sh and CFD-sh). A) heat map displaying all profiled metabolites in CO-Fibs and PH-Fibs (transfected with Scr-sh and CFD-sh). B) Enrichment pathway analysis using MetaboAnalyst (version 6.0), highlighting the top 25 enriched pathways in PH-Fibs Scr-sh compared to CO-Fibs, and matching these with the same pathways enriched in PH-Fibs CFD-sh compared to CO-Fibs.

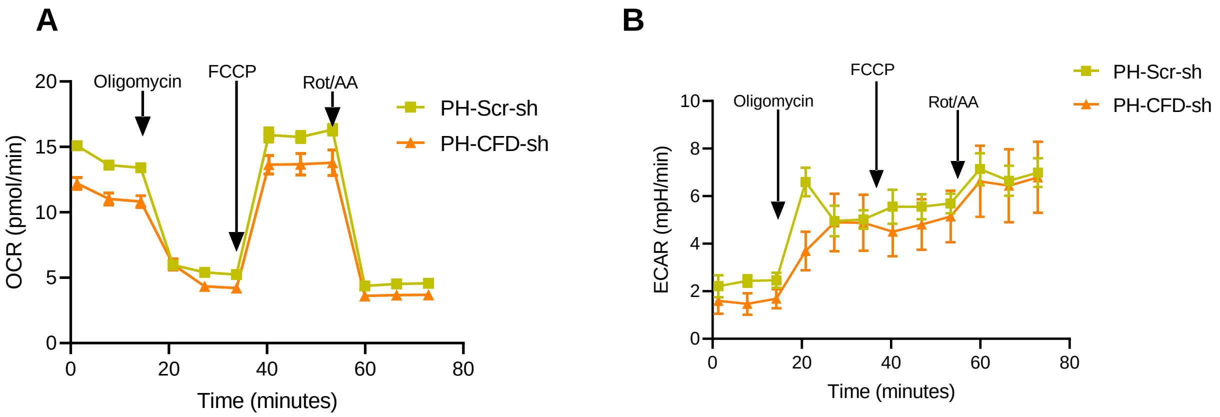

Supplementary Figure 5. Mitochondrial respiratory function parameters of PH Fibs transfected with Scr-sh and CFD-sh were assessed using the Seahorse XFe96 extracellular flux analyzer. A, B) Results showed the kinetics of the oxygen consumption rate (OCR) and extracellular acidification rate (ECAR) both at baseline and following treatments with oligomycin, FCCP (Carbonyl cyanide-p-trifluoromethoxyphenylhydrazone), and Rot/AA (Rotenone and Antimycin A)

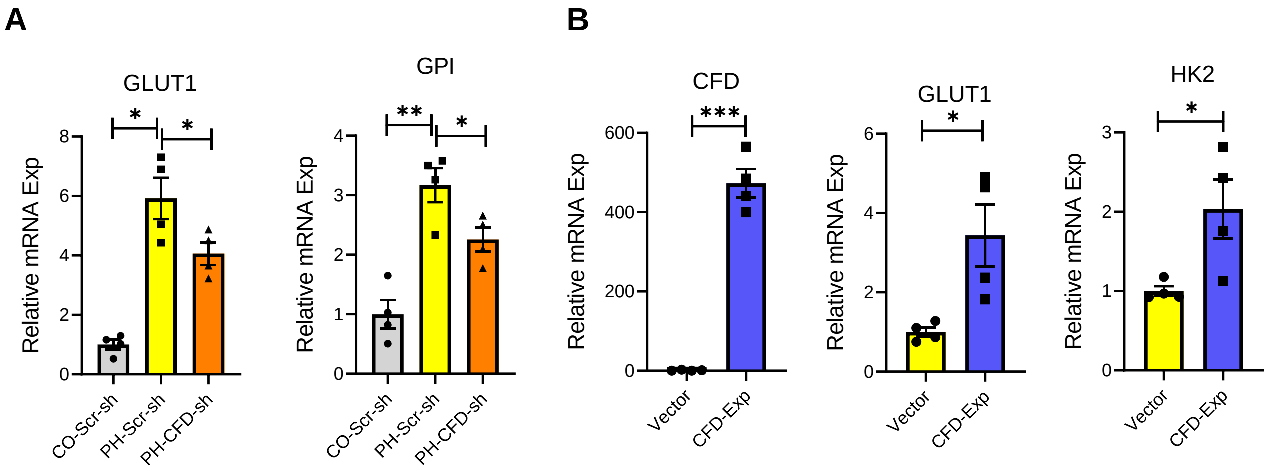

Supplementary Figure 6: The expression of CFD positively influences the expression of metabolic genes in adventitial fibroblasts.

A) The qRT-PCR analysis of GLUT1 and GPI in CFD knockdown PH-Fibs showed a decrease in their expression. B) In contrast, the mRNA expression levels of GLUT1 and HK2 increased significantly with CFD overexpression in PH-Fibs. A paired 2-tailed t-test was used to compare two groups of samples and for comparisons involving more than two groups with one variable, a one-way ANOVA followed by a Holm-Sidak post-test was used. Data are presented as mean ± SEM from bovine control (Scr-sh): n=4, PH fibroblast (Scr-sh, and CFD-sh): n=4 PH fibroblast (Vector): n=4 and PH fibroblast (CFD overexpression): n = 4, p-value ≤ 0.05 indicates a significant change. *P ≤0.05, **P < 0.01, ***P < 0.001.

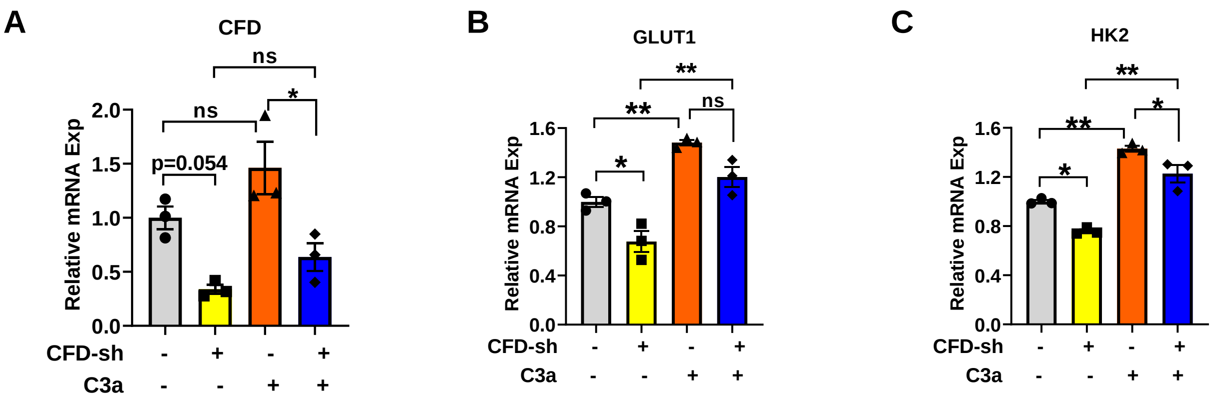

Supplementary Figure 7: Exogenous C3a peptide treatment in PH-fibroblasts reversed the effects of CFD knockdown on metabolic genes. A, B, and C) qRT-PCR data of CFD knockdown PH-Fibs treated with C3a peptide (50 nM concentration) show that exogenous C3a peptide significantly reverses the effects of CFD knockdown on GLUT1 and HK2. For comparisons involving more than two groups with one variable, a one-way ANOVA followed by a Holm-Sidak post-test was used. Data are presented as mean ± SEM from PH-Fibs (Scr-sh and CFD-sh): n = 4 (technical replicates), p-value ≤ 0.05 indicates a significant change. *P ≤0.05, **P < 0.01.

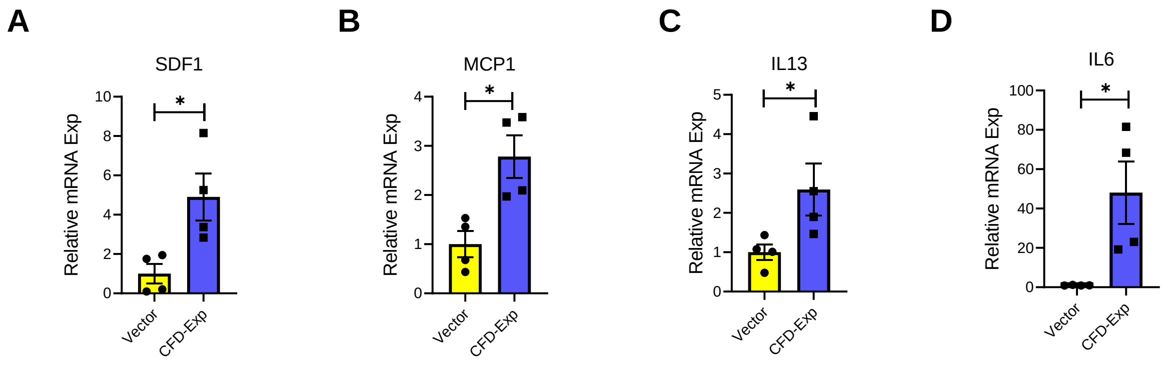

Supplementary Figure 8. CFD overexpression in PH-Fibs promotes the expression of pro-inflammatory genes. CFD overexpression promotes the expression of SDF1, MCP1 IL13 and IL6 in PH-Fibs. Unpaired 2-tailed t-test was used to compare two groups of samples. Data are presented as mean ± SEM from bovine control: n=4 and PH fibroblast (CFD overexpression): n = 4, p-value ≤ 0.05 indicates a significant change. *P ≤0.05.

Supplementary table 1

	Bovine qRT-PCR Primers	
**Gene**	**Forword Sequence**	**Reverse Sequence**
CFB	CTCTGGAGGGAGTAGAGATCAA	GAAGCCAGAAGGACACAAGTA
C3	AGGTCCAACTAGAGCCTGAA	CTGGCAATGATGTTGACCTTC
C3aR1	TGACCTACACTCACAGCCCT	TTGCATCTTTAGGCCAGCCA
CFD	CACCATCACTGAGCGAATGA	CAGATCCGTGAACCCGAGGT
IL6	GATGAAGCAGCAAGGAGACA	ATCCGTCCTTTTCCTCCATT
MCP1	CGCCTGCTGCTATACATTCA	ACACTTGCTGCTGGTGACTC
IL13	TGATCAGCATCTCCAACTGC	GAGGGCTTGTGAGGACAGAG
IL33	GTAAACCTGAGCCCCACAAA	AGGTTTGTTCTGGCAACTGG
GLUT1	GCTCATGGGCTTCTCAAAAC	CACGTACATGGGCACAAAAC
HK2	CGGAGCTCAACCACGACCAA	TGGTGGCTCCAAGCCCTTTC
ENO1	CCAAGTACAACCAGATCCTCAG	CATCAGATGCAGGTCTAAGGAG
GPI	GGACTCACATTGCCAAAACG	AAACCACTCCTTCGCTGTC
ACO1	TGGTTCTGTGGTAATCGCTG	AGGAGACAGGCTAGTTTTGATG
SDF1	TCCAATCCCTCCACATTCTC	TGAGCTGGCTTTTGAAGGAT
	**Human qRT-PCR Primers**	
**Gene**	**Forword Sequence**	**Reverse Sequence**
CFB	GGGACACGAGAGCTGTATGG	TTCTATCTCCAGGTCCCGCT
C3	GGAAGGACACTTGGGTGGAG	AACCATGCTCTCGGTGAAGG
C3aR1	CTCCATGGTCATTCTCAGCCT	ACCCACAGCACCAGCCCATT
CFD	GACACCATCGACCACGAC	GTTGACTATGCCCCAGCC
SDF1	TCGGTGTGCACCAGGGTTGA	TGCTGGGCTTGGCCCTAGTT
HK2	CCTTCCCTGAACCTTTTCCA	TGCTAGACACCAGACTCCAA
GLUT1	TGCCACCATTGGCTCCGGTA	AGCAGTGCTAGCGCGATGGT
IL6	ACAAGCGCCTTCGGTCCAGT	TGTGTGGGGCGGCTACATCT

Supplementary Table 2:

	**Target sequence**	**Guide sequence**
Scramble_shRNA	CCTAAGGTTAAGTCGCCCTCG	CGAGGGCGACTTAACCTTAGG
CFD-shRNA1	TGCTGCAGCTCTCTGAGAAAG	CTTTCTCAGAGAGCTGCAGCA
CFD-shRNA2_F	CACCATCACTGAGCGAATGAT	ATCATTCGCTCAGTGATGGTG
