# Supplementary material for: An intracellular complement system drives metabolic and proinflammatory reprogramming of vascular fibroblasts in pulmonary hypertension
Source: JCI Insight. 2025 Feb 13;10(6):e184141. doi: 10.1172/jci.insight.184141 (PMC11949053; doi:10.1172/jci.insight.184141)

Figure 2: Human IPAH fibroblasts exhibit elevated expression of complement genes (C3, CFD, and CFB) and increased production of activated C3 fragments (C3d and C3a).

B

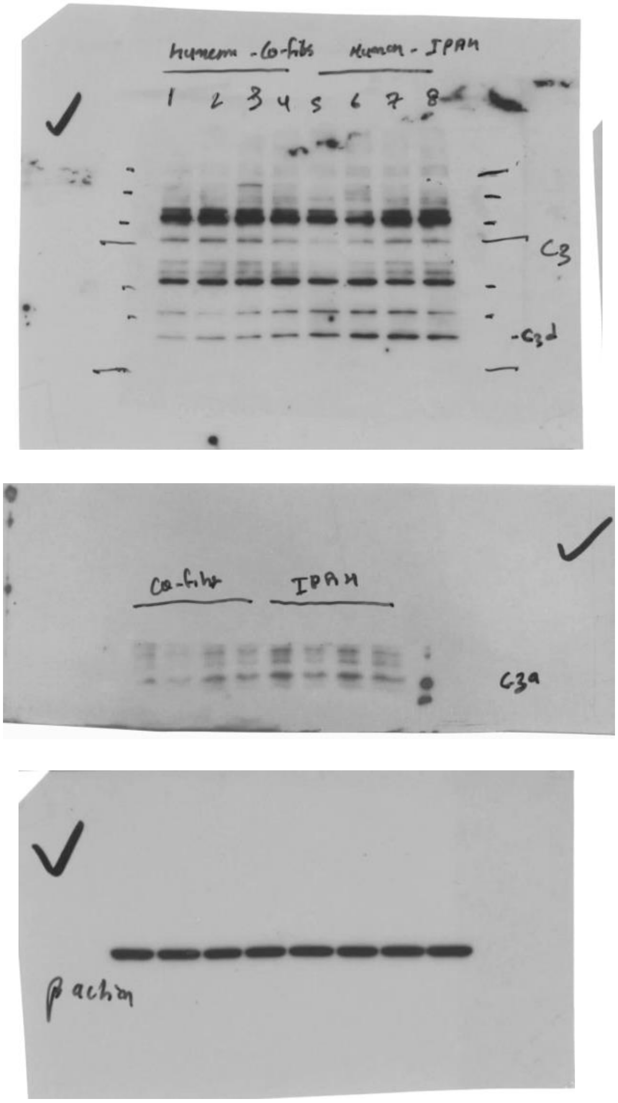

E

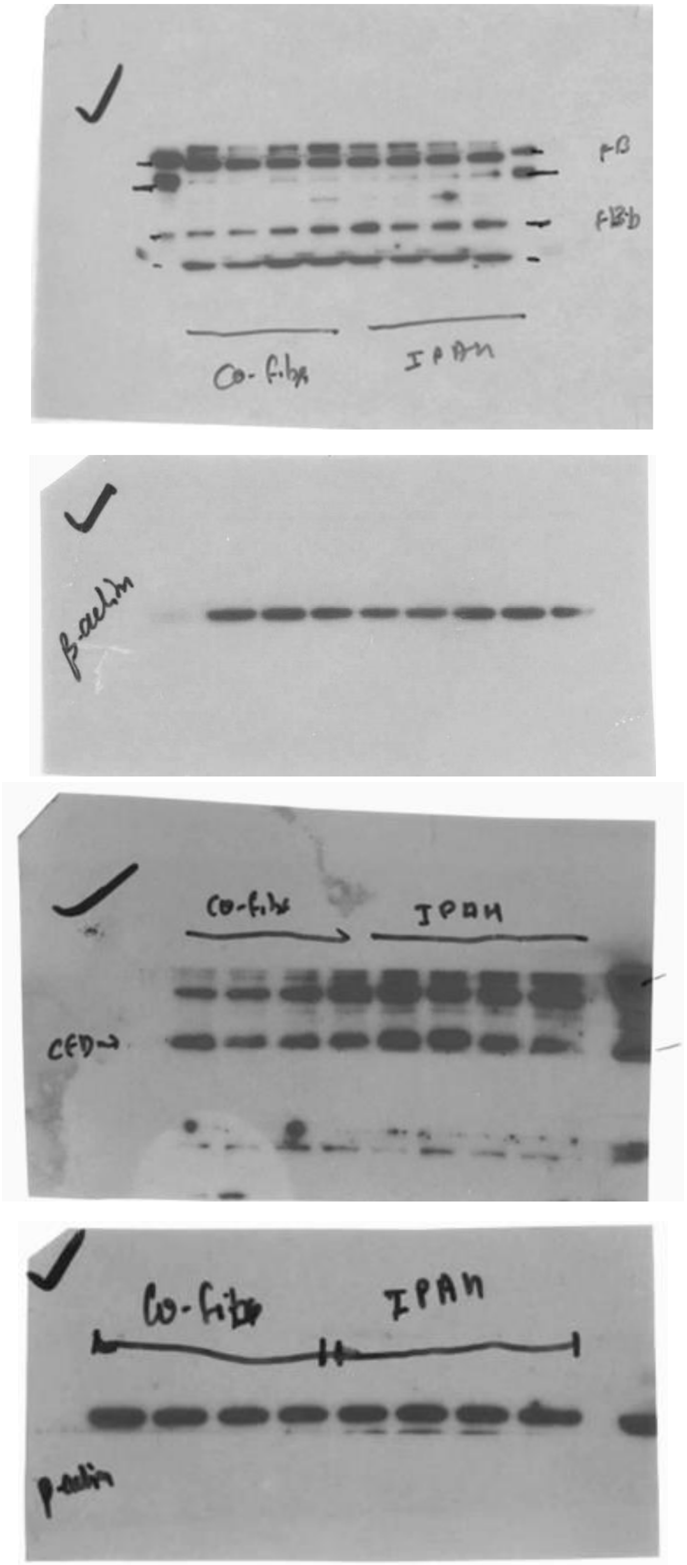

Figure 3: Bovine fibroblasts show increased expression of complement genes (C3, CFD, and CFB) and increased production of activated C3 fragments (C3d and C3a).

B

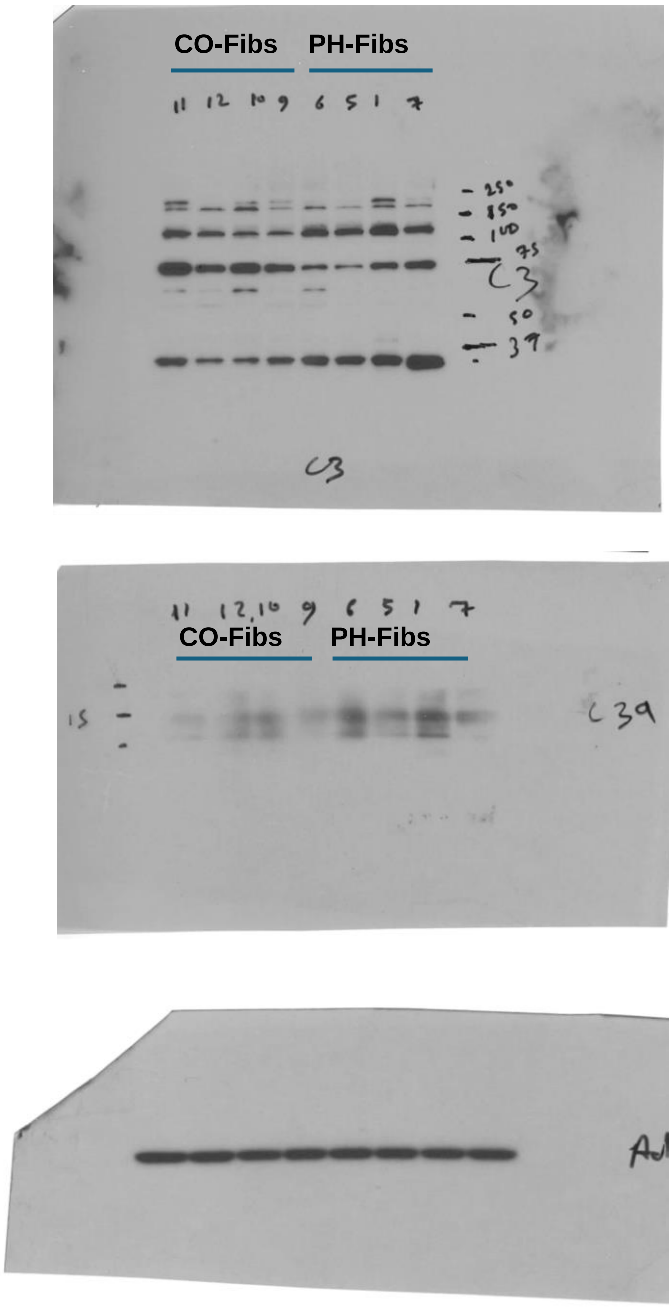

E

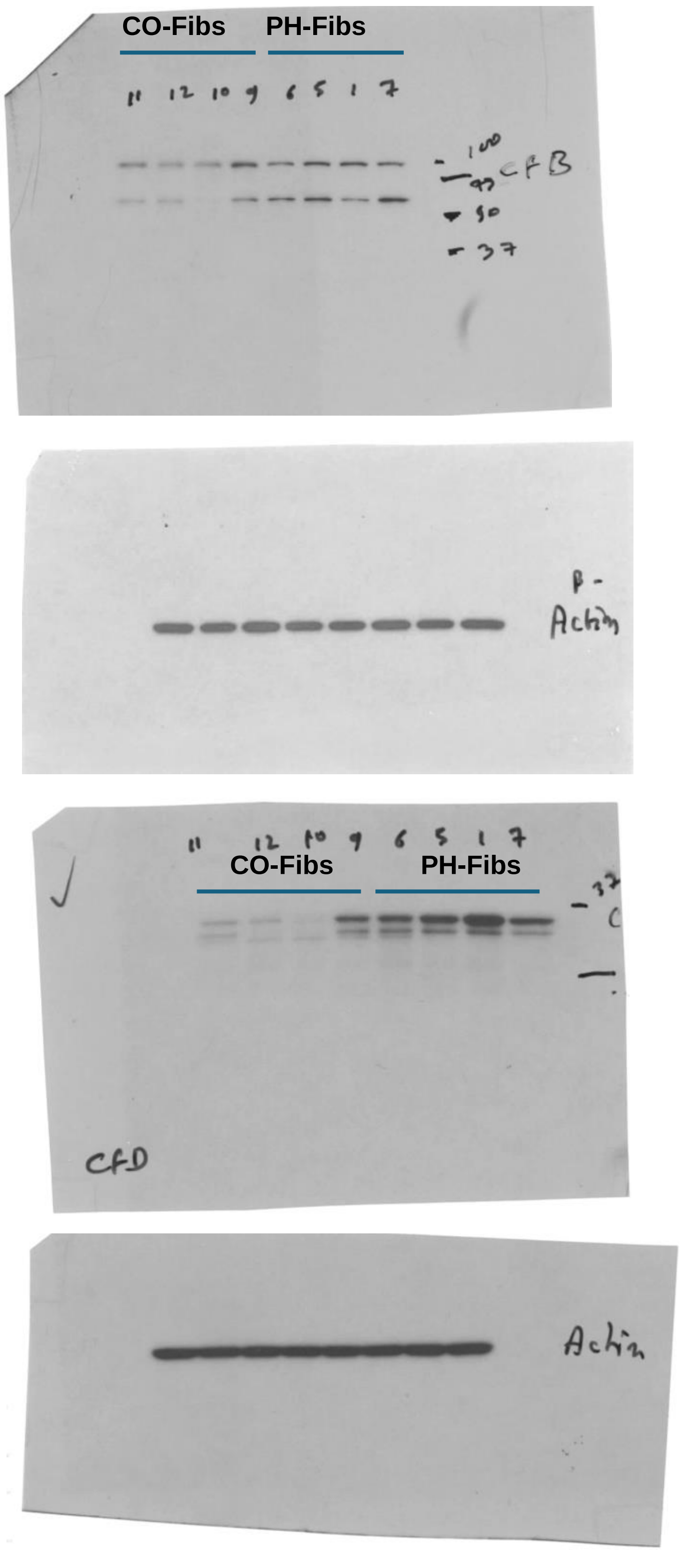

Figure 4: CFD regulates the activation of C3 in bovine pulmonary artery adventitial fibroblasts.

B

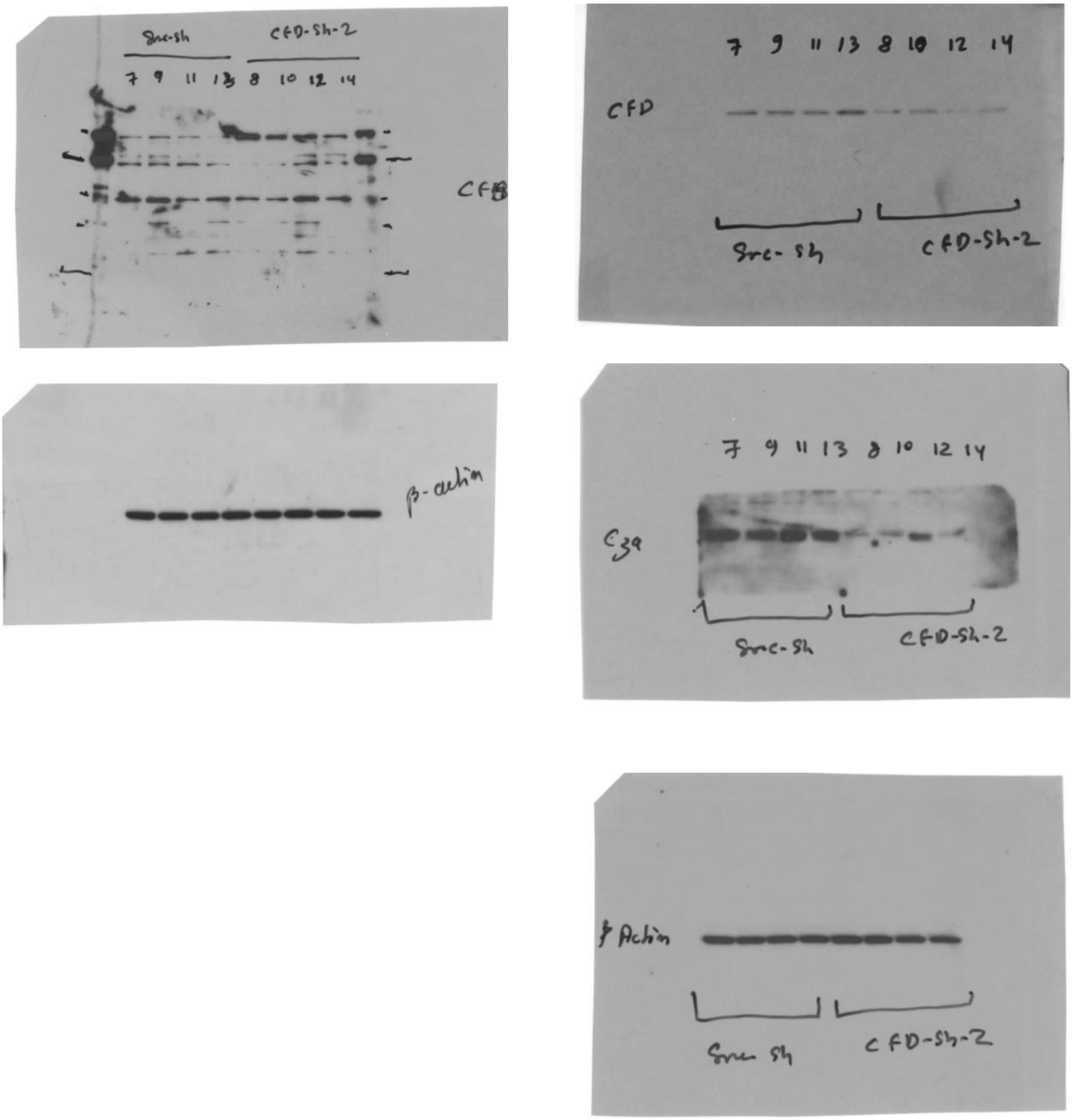

Figure 4: CFD regulates the activation of C3 in bovine pulmonary artery adventitial fibroblasts.

E

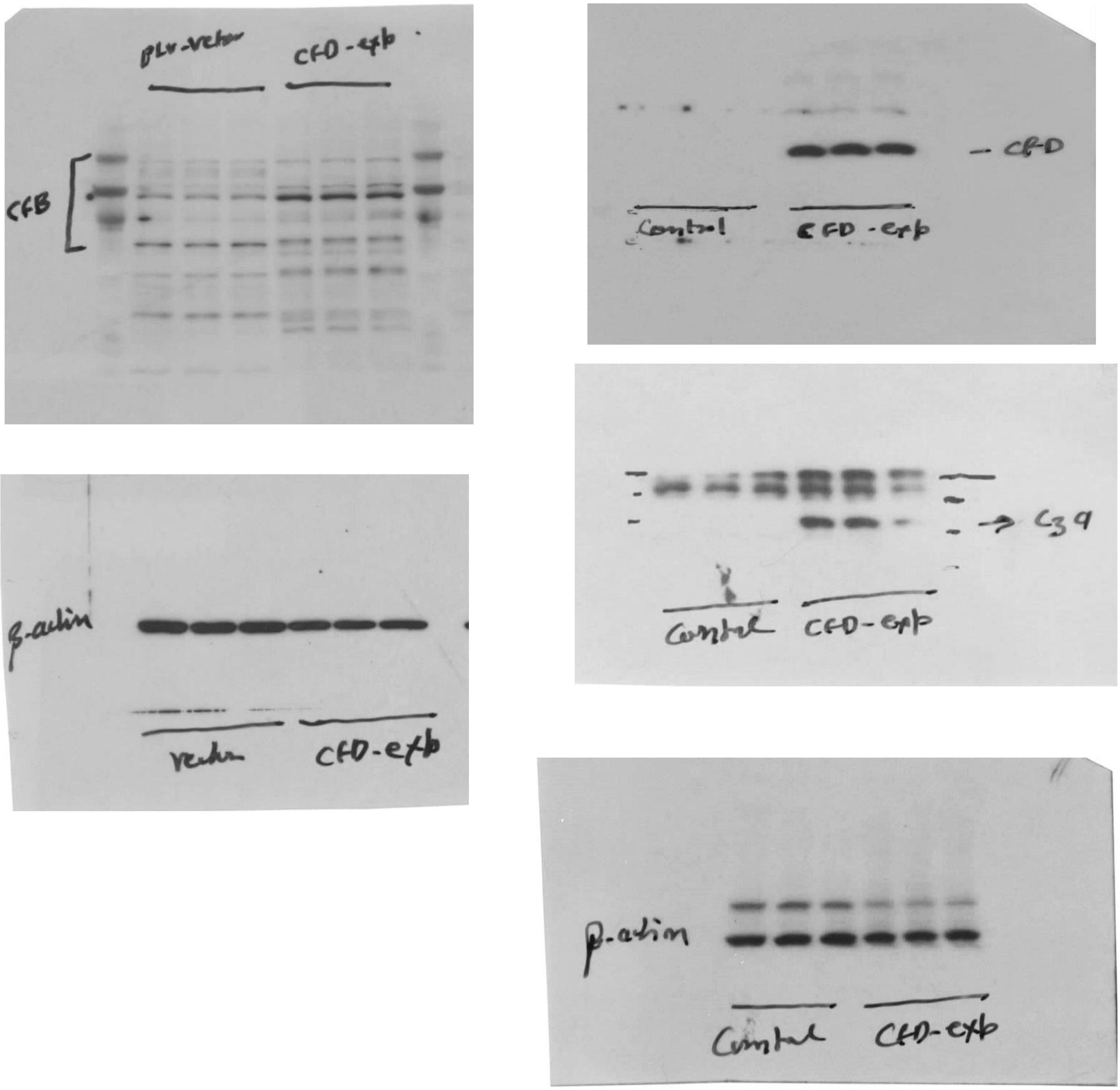

Figure 5: CFD is localized in the cytoplasmic region of adventitial fibroblasts and interacts and activates CFB (CFBb)

A)

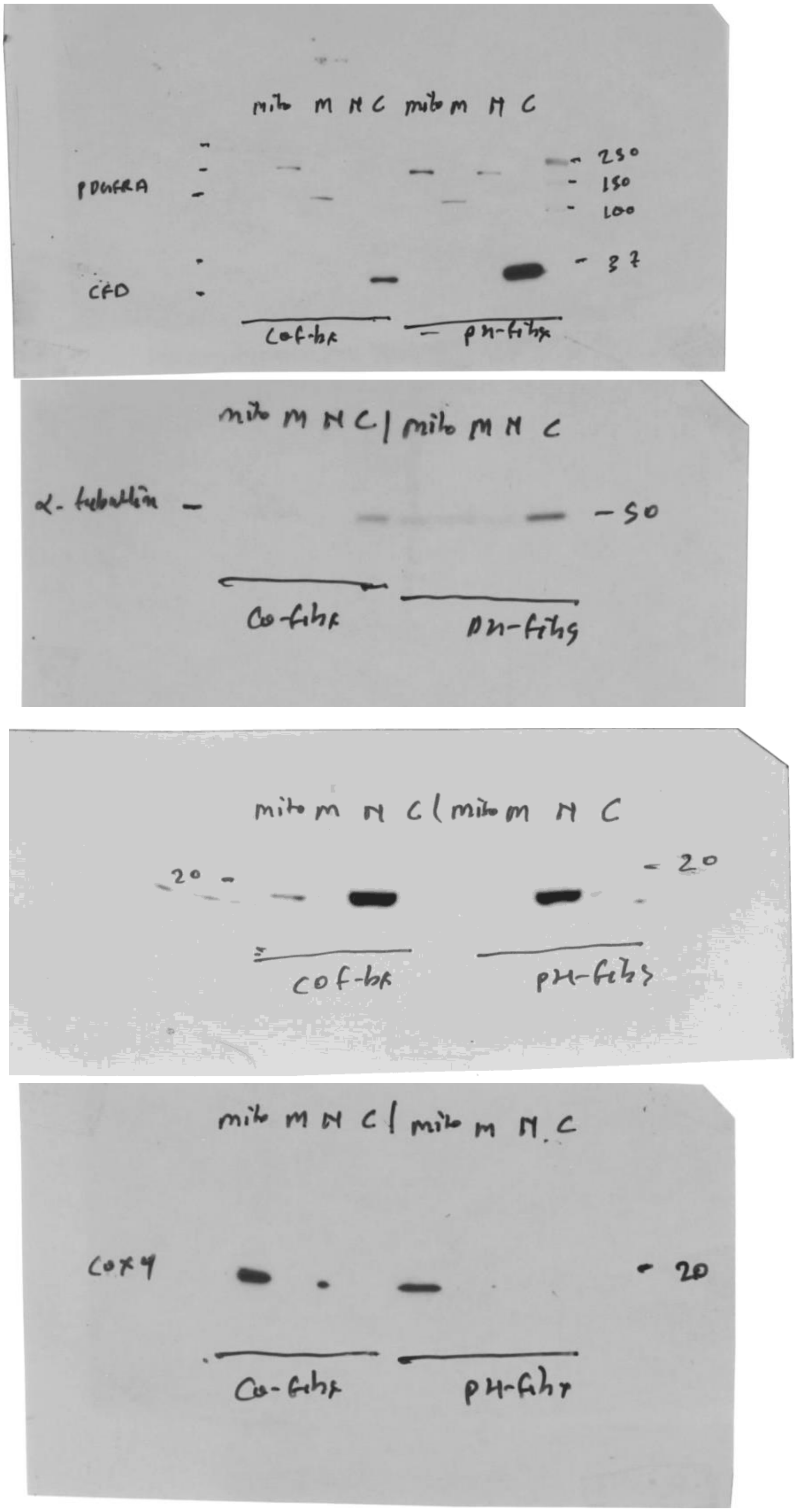

Figure 5: CFD is localized in the cytoplasmic region of adventitial fibroblasts and interacts and activates CFB (CFBb)

B)

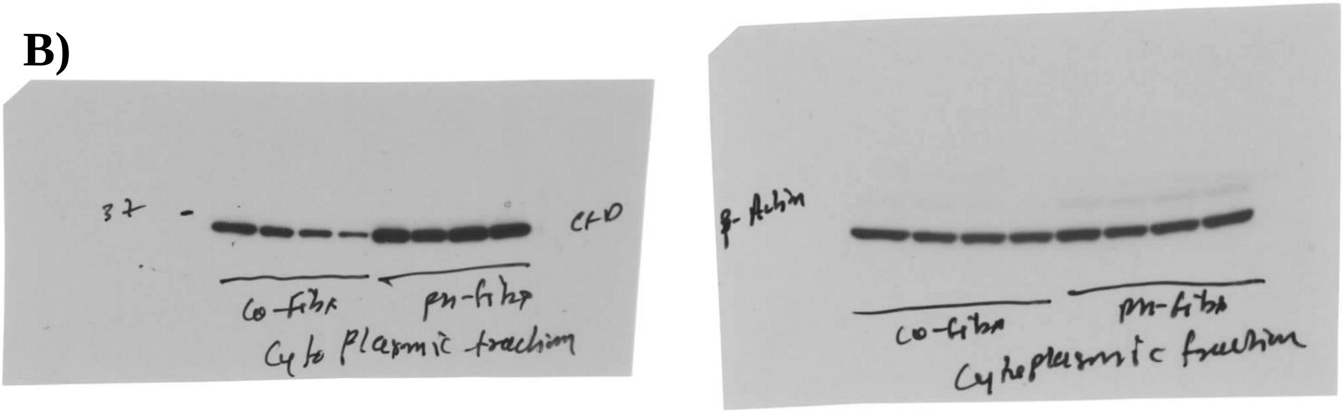

C)

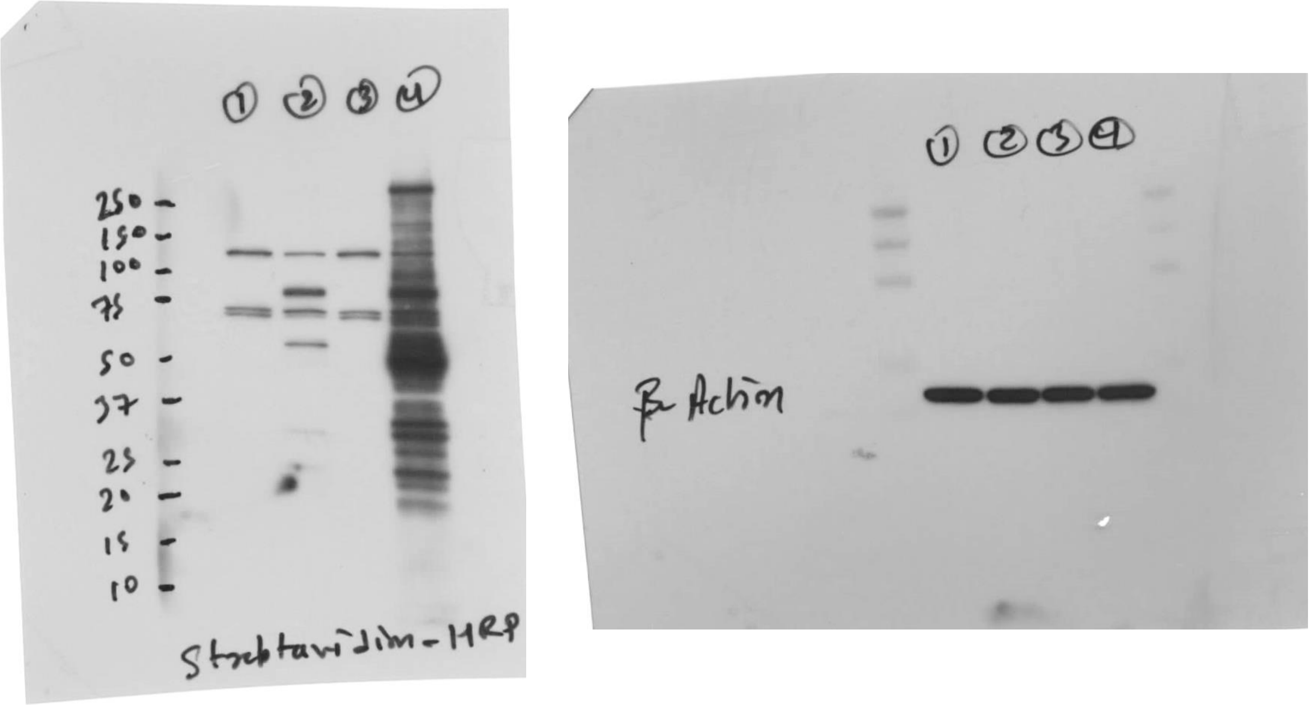

Figure 5: CFD is localized in the cytoplasmic region of adventitial fibroblasts and interacts and activates CFB (CFBb)

D)

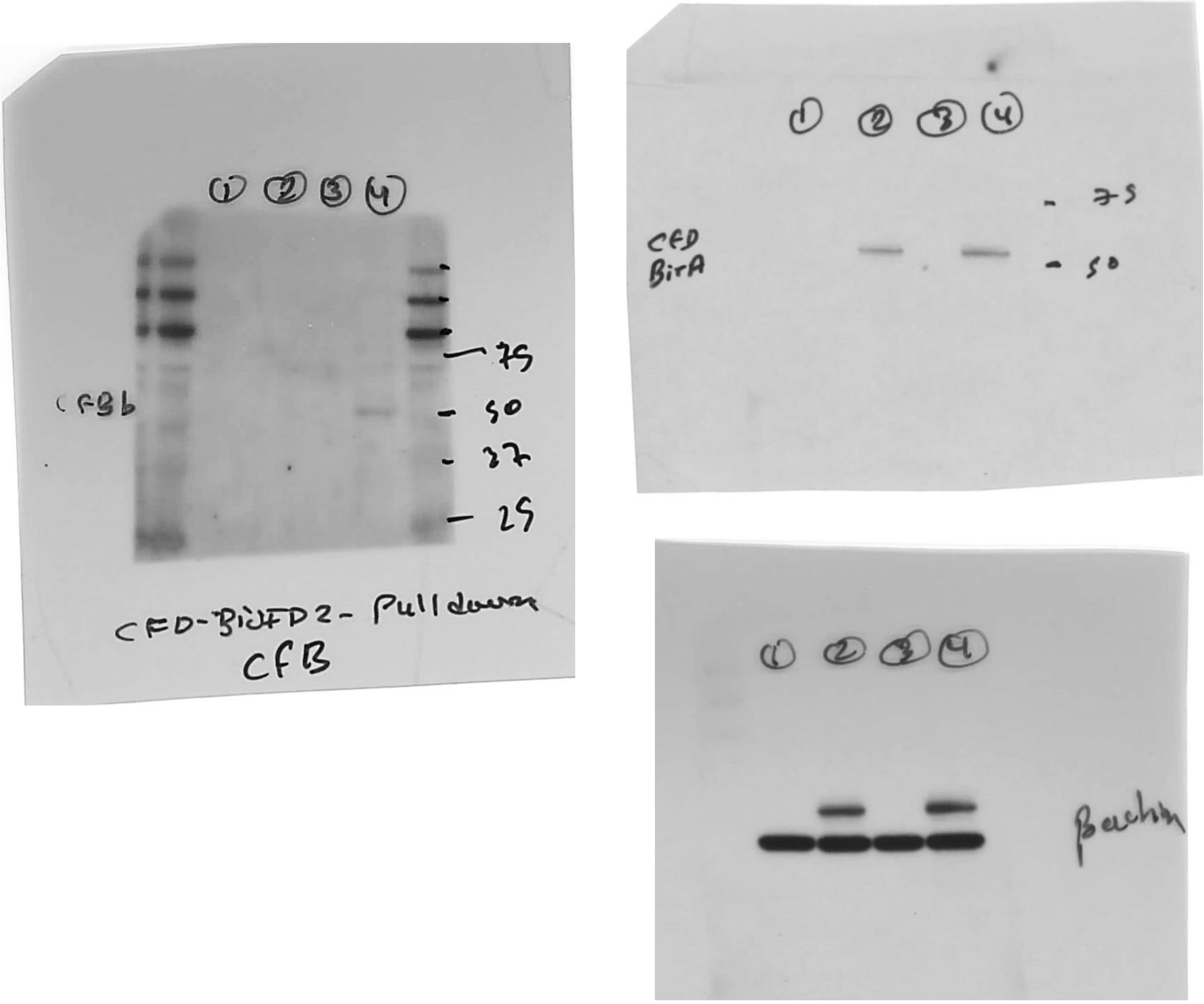

Figure 6. C3aR1 expression is localized on the cell membrane and mitochondria in pulmonary vascular fibroblasts.

B

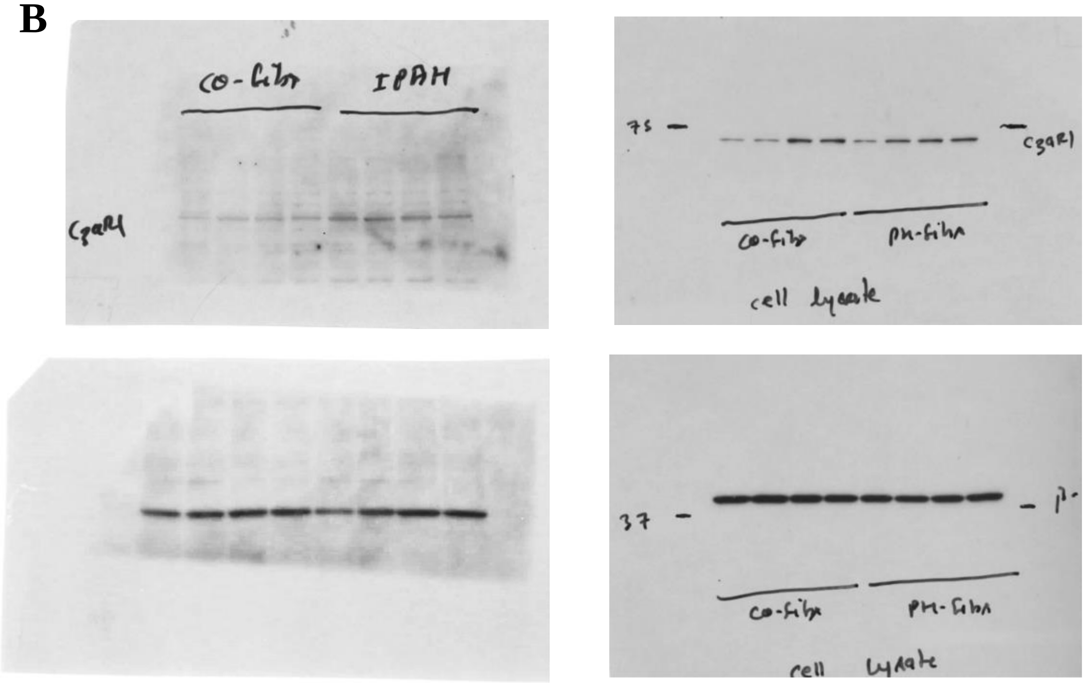

Figure 6. C3aR1 expression is localized on the cell membrane and mitochondria in pulmonary vascular fibroblasts.

D

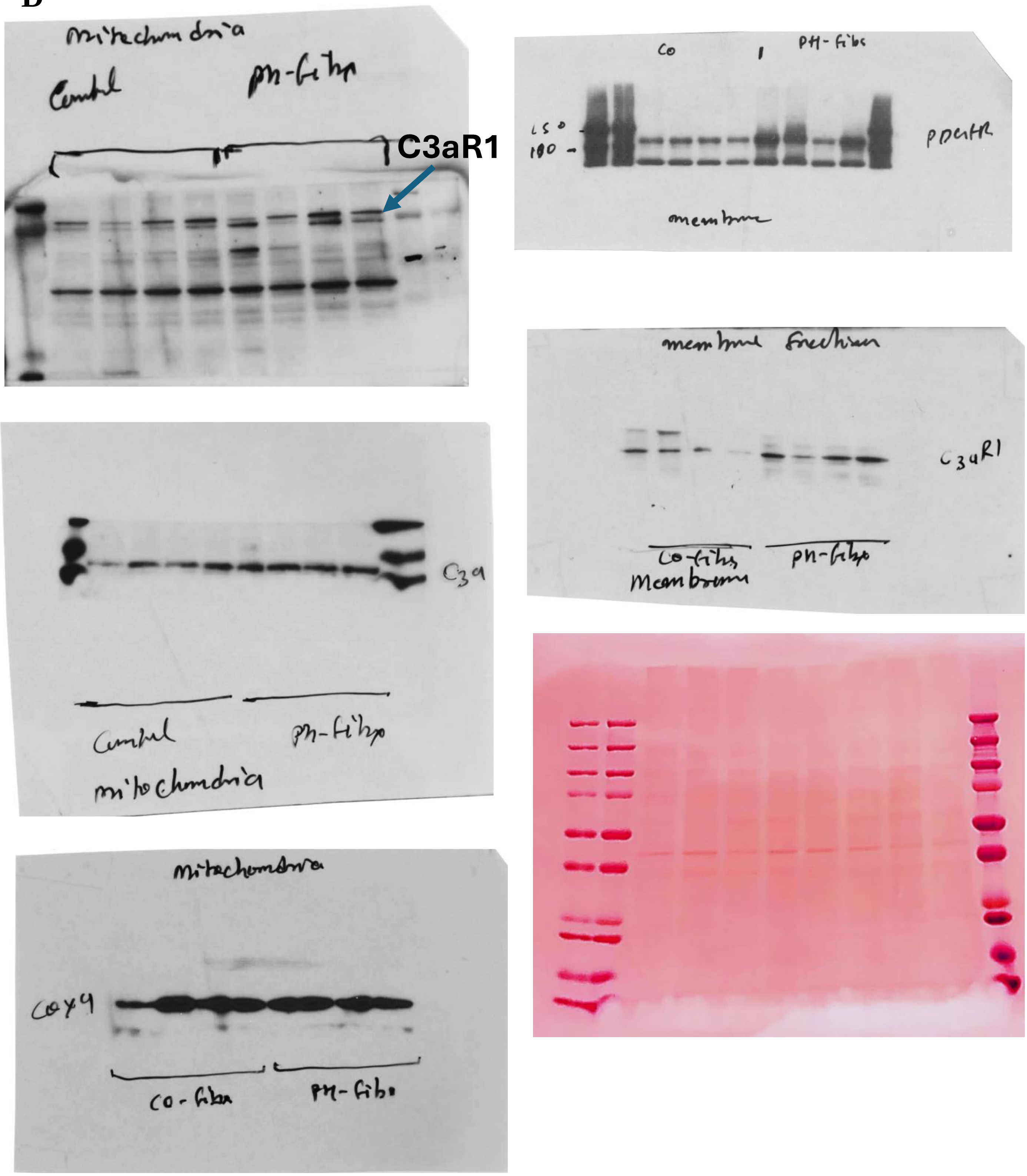

Supplementary Figure

Supplementary Figure 2: CFD inhibitor (Vemircopan) treatment reduced the activation of CFB and C3 (C3a) in PH-Fibs.

A)

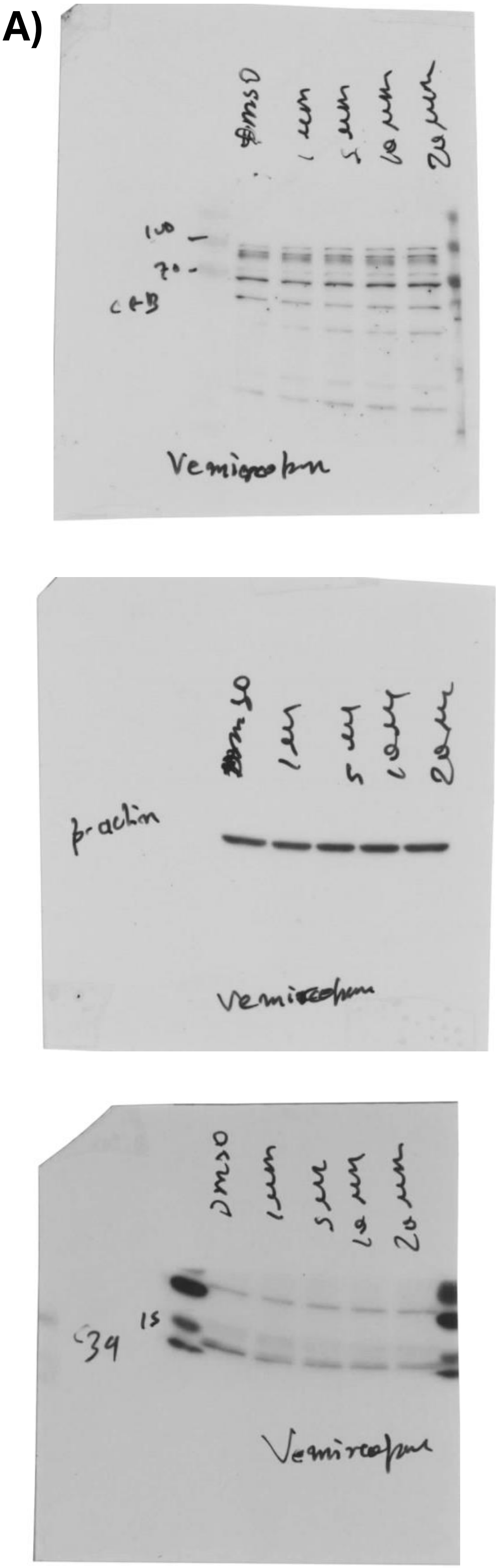

B)

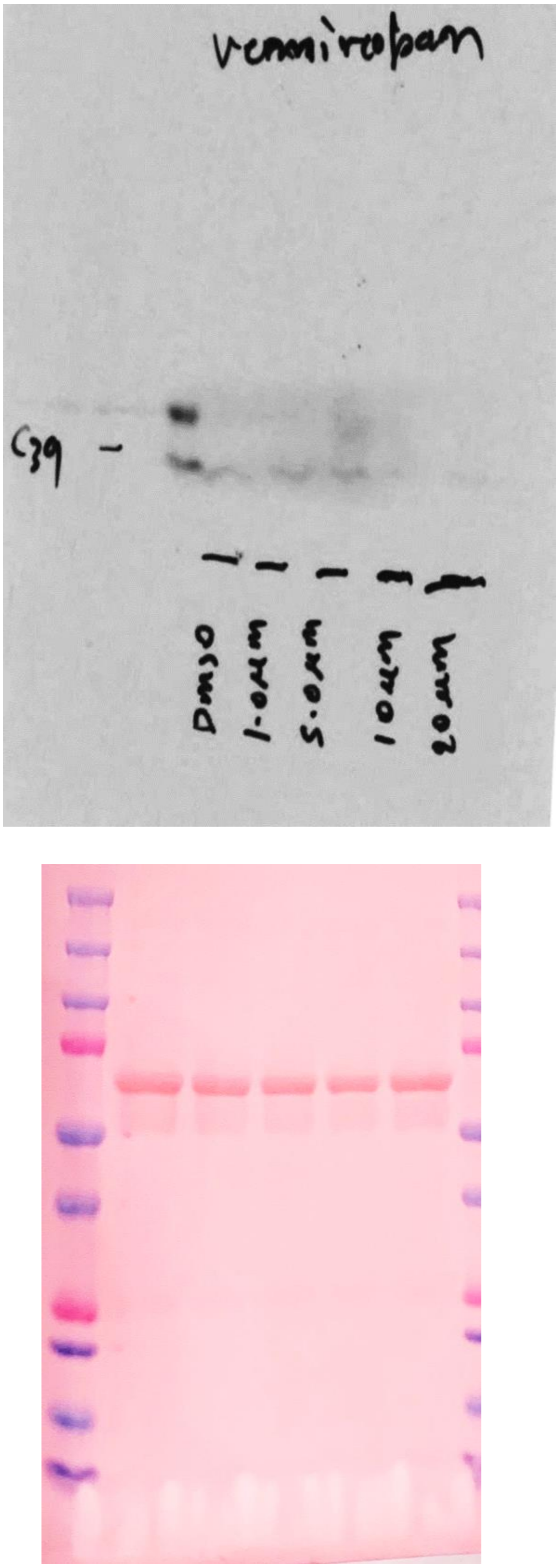

Supplement: Unedited blot and gel images [file jciinsight-10-184141-s253.pdf]
